# Microbial contamination and antimicrobial resistance in dried fish from informal markets in Gauteng Province, South Africa: A One Health food safety assessment

**DOI:** 10.14202/vetworld.2026.3716-3741

**Published:** 2026-08-27

**Authors:** Siphiwe Rendy Nkosi, Mpinda Edoaurd Tshipamba, Ngoma Lubanza, B.G. Mokolopi, Mulunda Mwanza

**Affiliations:** 1 Department of Animal Health, School of Agriculture, Faculty of Natural and Agricultural Sciences, North-West University, Mafikeng Campus, Private Bag X2046, Mmabatho 2735, South Africa.; 2 Department of Agriculture and Animal Health, College of Agriculture and Environmental Sciences, University of South Africa, Florida Science Campus, Johannesburg, South Africa.

**Keywords:** antimicrobial resistance, dried fish, food safety, informal markets, microbial contamination, One Health, public health, South Africa

## Abstract

**Background and Aim:**

Dried fish sold in informal markets is an affordable source of protein but may be vulnerable to microbial contamination because of inadequate hygiene and handling practices. This study aimed to assess microbial contamination levels, identify bacterial isolates using 16S rRNA sequencing, and determine the antimicrobial resistance (AMR) profiles of bacteria recovered from sun-dried, smoked, and salted fish sold in informal markets in Gauteng Province, South Africa. The research addressed limited information on the microbiological quality and AMR of dried fish sold in South African informal markets, particularly the integration of culture-based, molecular, and resistance-profiling approaches in a One Health framework.

**Materials and Methods:**

A total of 140 dried fish samples (80 sun-dried, 40 smoked, and 20 salted) were collected from 12 informal markets in Johannesburg and Pretoria using convenience sampling. Total bacterial counts (TBC) were determined on nutrient agar, followed by phenotypic characterization and molecular identification using *16S rRNA* gene sequencing. Antimicrobial susceptibility testing was performed using the disk diffusion method against seven antibiotics. Data were analyzed using descriptive statistics, analysis of variance with Bonferroni correction, and chi-square tests, with statistical significance set at p < 0.05.

**Results:**

TBC ranged from 0.8 × 10⁷ to 5.34 × 10⁷ colony forming units/g, with a significant difference between sun-dried and salted fish after Bonferroni adjustment (p = 0.012). Sixty-seven bacterial isolates were identified, dominated by *Clostridium* spp. (10/67, 14.92%), *Staphylococcus* spp. (8/67, 11.94%), *Staphylococcus xylosus* (6/67, 8.95%), and *Klebsiella pneumoniae* (5/67, 7.46%), including *Clostridium botulinum* (2/67, 2.98%). High resistance was observed to streptomycin (73.1%) and erythromycin (58.2%), and several bacterial isolates exhibited multidrug resistance. No significant differences in bacterial counts were detected among sampling locations (p = 0.457).

**Conclusion:**

This study provides critical evidence of multidrug-resistant bacteria, including *C. botulinum*, in dried fish from informal markets, highlighting serious food safety and public health risks. Findings underscore the need for enhanced hygiene practices, vendor training, and regulatory oversight to mitigate contamination and the spread of AMR in informal food systems.

## INTRODUCTION

Informal markets serve as a critical source of affordable food for many South Africans [1, 2]. Nevertheless, poor infrastructure, inadequate sanitation, and limited regulatory oversight remain important challenges in informal market in Gauteng Province, South Africa. Sun-dried, salted, and smoked fish are regularly sold in open-air markets where exposure to environmental contaminants is high, and improper storage and handling elevate the risk of microbial contamination [3]. These Informal markets are an integral part of urban life in low- and middle-income countries, including South Africa, due to high unemployment rates, cultural food preferences, and limited opportunities [4, 5]. Despite the widespread availability and consumption of street-vended foods, scientific data on their microbiological quality and safety in South Africa remain limited [6]. Concerns regarding food safety in informal markets have therefore prompted calls for improved regulation and hygiene [7]. In particular, information on the microbiological quality of dried fish sold in South African informal markets remains limited. Research and observation have identified food safety issues linked to street vendors operating in unclean work areas [8]. Although other research has examined microbial contamination of dried fish in other African nations, including Nigeria, Ethiopia, and Zimbabwe, information remains limited in South Africa, particularly in informal urban markets in Gauteng Province. Such markets are a vital source of food for low-income communities, and no significant research has yet been conducted on their microbiological safety and antimicrobial resistance (AMR) profiles. Additionally, few studies have formally assessed the microbiological quality and associated public health risks of dried fish within informal food systems.

While informal markets offer food security and good livelihood opportunities, quality food products may pose problems of safety due to poor infrastructure, sanitation and regulatory control. Hence, strengthening the informal Food Trading Program may improve hygiene and food-handling practices and reduce the risk of foodborne diseases among consumers [9]. These informal markets offer affordable food to urban populations in South Africa [10] and continue to play a significant socioeconomic role, particularly for undereducated and marginalized communities [11, 12]. Poor sanitation practices in these markets, along with improper waste disposal and limited access to clean water, may increase the risk of microbial contamination and foodborne pathogens [13]. Dried fish may be contaminated with potentially pathogenic bacteria, including *Escherichia coli*, *Salmonella* spp., and *Staphylococcus aureus,* during post-harvest handling, drying techniques, and packaging [4]. Although dried fish processing and preservation methods such as sun-drying, smoking, and salting may reduce bacterial growth, they do not completely eliminate contamination risks [15]. Previous studies [16, 17] have reported foodborne bacteria, including *S. aureus*, *Salmonella,* and *E. coli,* on street-vendor-sold dried fish, indicating improper cleaning and storage practices that increase food contamination risks. The Food and Agriculture Organization, alongside the World Health Organization [18], confirmed [19, 20] that contaminated food causes serious health problems globally since foodborne germs remain significant agents of illness and death in low- and middle-income nations. Dried fish serves as an essential protein source in low- and middle-income countries, but it is contaminated by substandard handling practices and inadequate processing and storage methods. The growth of foodborne bacterial AMR represents a critical global health threat, especially for populations in low- and middle-income countries (LMICs). The misuse of antibiotics in aquaculture and post-harvest practices directly contributes to the emergence of drug-resistant pathogens isolated from dried fish products [21, 22]. A particular concern in LMICs is the emergence of antimicrobial-resistant foodborne bacteria, where antimicrobial stewardship, surveillance, diagnostic capacity, and regulatory systems might be limited. [23, 24]. Additionally, insufficient monitoring of AMR continues to affect South Africa's informal market, underscoring the need to strengthen surveillance programs [25, 26]. Most dried fish-based food products exhibit resistance to standard antibiotic medications, including cephalosporins, fluoroquinolones, and macrolides [27]. On the other hand, researchers have documented antibiotic resistance in *Salmonella* spp., *E. coli,* and *S. aureus*. The rise of drug-resistant bacteria is mainly linked to the uncontrolled use of antibiotics in fish farming, poor sanitation, and a lack of diagnostic tools, which are major problems for low- and middle-income countries, making it hard for them to manage and combat the spread of AMR [28, 29]. In this study, the sun dried, smoked, and salted fish were used since they are some of the most prevalent and popular types of preserved fish available in the informal markets in Gauteng Province. This study applied a comprehensive method comprising hygiene observations at vending points, culture-based microbiological analysis, molecular identification by *16S rRNA* gene sequencing, and antimicrobial susceptibility testing. This strategy allowed assessment of the microbiological quality of dried fish alongside vending-site hygiene conditions and the antimicrobial susceptibility patterns of the recovered bacterial isolates, from a One Health perspective.

The detection and prevention of foodborne pathogens have advanced with improvements in accuracy and efficiency [30]. Furthermore, nucleic acid-based techniques offer high specificity for detecting harmful bacteria in food [31, 32]. However, improving hygiene and food-handling measures and practices is critical to reducing contamination and the dissemination of drug-resistant pathogens through the food chain, thereby reducing the risk of foodborne outbreaks involving drug-resistant bacteria [13]. Therefore, this study aimed to evaluate the food safety practices and microbiological quality of dried fish, molecularly characterize bacteria isolated from dried fish sold in informal markets in Gauteng Province, South Africa, and determine their antimicrobial susceptibility.

## MATERIALS AND METHODS

### Ethical approval

The study protocol was reviewed and approved by the Health Research Ethics Committee of North-West University, South Africa, under Approval No. NWU-01880-19-A5 (Risk Category 2).

The research involved observational assessment of food handling and hygiene practices at informal market vending sites and the collection of dried fish samples for microbiological and molecular analyses. Human participation was limited to vendors who voluntarily provided information regarding their vending practices and consented to site observations. Verbal informed consent was obtained from all participating vendors before data collection after explaining the objectives, procedures, and voluntary nature of the study. Participants were informed of their right to decline to participate or withdraw at any stage without consequences.

No personal identifiers or confidential information were collected or reported, and all observations and data were anonymized to protect participant privacy. The study did not involve clinical interventions, biological sampling from humans or animals, or procedures causing physical or psychological harm. Dried fish samples were purchased through routine commercial transactions from informal markets, and sample collection did not interfere with vendors' normal business activities. All laboratory procedures involving bacterial isolation, molecular characterization, and antimicrobial susceptibility testing were conducted in accordance with the institutional biosafety guidelines and standard microbiological laboratory practices at North-West University. The study complied with applicable institutional and national ethical requirements governing the research.

### Study period and location

A cross-sectional descriptive study was conducted in Gauteng Province, South Africa, between September 2019 and July 2020. This sampling period was chosen to capture the effects of varying environmental factors and market forces on drying processes, storage procedures, and microbial contamination rates. Sun-dried, salted, and smoked fish were purchased from different unofficial marketplaces within Johannesburg and Pretoria. Information was collected from Sunnyside, Yeoville, Rosettenville, and MTN taxi rank markets of Johannesburg.

### Study design

Johannesburg and Pretoria, located in Gauteng Province, South Africa, were the two study areas. A cross-sectional descriptive study was conducted in Gauteng Province, South Africa, between September 2019 and July 2020. This sampling period was chosen to capture the effects of varying environmental factors and market forces on drying processes, storage procedures, and microbial contamination rates. To determine the microbiological quality of dried fish. Sun-dried, salted, and smoked fish were purchased from different unofficial marketplaces within Johannesburg and Pretoria. Because informal markets are dynamic, convenience sampling was used to select vendors. The targeted informal markets were large stores with high client volume and several vendors selling the same product. Information was collected from Sunnyside, Yeoville, Rosettenville, and the MTN taxi rank markets of Johannesburg. The categorization of the dried fish samples into sun-dried, smoked, and salted was mainly done based on the declaration of the vendors at the point of purchase, with some form of visual observation of the product. The processing method was confirmed by characteristics such as texture, color, the presence of salt crystals, and, where necessary, smoke. 

### Observation study

The structured checklist was adapted from published research and was used to evaluate the vendors' hygiene behaviors and personal hygiene practices at the informal market vending [13]. Observations were conducted during sample collection. To monitor subsequent processes, the observation sheet focused on food service management, including general hygiene of street vendors, food storage, food packaging, handling, waste, and hygiene practices. The study also evaluated fly prevalence, the presence of stagnant water and insoluble materials, food vendors’ personal hygiene (hands, nails, and hair), protective clothing worn by food vendors, and food protection measures taken by food vendors at the vending site. To contextualize the hygiene practices applied by vendors and observed during sample collection, basic vendor demographic information, such as age range and gender, was recorded.

### Vending site and surroundings

The situation of the presence of stagnant water around the vending site was investigated:

1. Existence of insects and flies

2. The state of neatness or cleanliness of the vending site. If any of these characteristics were identified at the point of sample collection, a tick was placed on the checklist. The hygienic measures used by the vendors were based upon:

3. Hand washing before touching the dried fish

4. Cleaning of hands.

5. Washing hands with disinfectants.

6. Weighing dried fish in plastic bags.

7. Storage method of dried fish.

### Sample size determination, collection and microbiological analysis

A non-probability sampling method (convenience sampling) was used due to the dynamic nature of informal markets in Gauteng Province and because the population of dried fish vendors was unknown [7, 8]. The sample size (n = 140) was determined based on feasibility and the representativeness of the sampled informal markets. Because no prior data exist on microbial contamination of dried fish in informal markets in Gauteng, the study was descriptive, and the sample size was deemed adequate to determine variation by fish type and sampling site. The study collected 140 samples during visits to 12 markets. The same markets were used to obtain samples on two occasions. The first phase focused on collecting almost 70 dried fish samples, with the remaining 70 collected a month later. The target markets were the most dynamic informal trading markets within Gauteng Province and were selected based on high customer flow, fish sales volume, and availability. These markets have high numbers of informal vendors supplying low-cost food products to local populations. Informal vendors participated in this study based on their availability and willingness at the time of sampling, as there is no formal list of dried fish vendors in such informal environments. In the present study, the collected samples were classified based on the techniques used to preserve them. Three types of dried fish were collected: salted (20), sun-dried (80), and smoked (40). For identification, samples of the collected fish were placed in zip-lock plastic bags, marked with a permanent marker, and kept in a cooler box with ice packs at approximately 4 °C for 2 hours before transport to the Northwest University Animal Health Department for further analysis. Table 1 summarizes sample collection.

**Table 1:** Overall sampling collection.

**Location**	**City**	**No. of markets**	**Dried fish samples collected**	**Approximate coordinates**	**Environmental characteristics**
Sunnyside	Pretoria	5	50	–25.7544, 28.2076	High-density residential area, street vending, heavy pedestrian activity
Yeoville	Johannesburg	3	30	–26.1839, 28.0642	Informal trading hub, mixed residential-commercial area, high human traffic
Rosettenville	Johannesburg	2	30	–26.2545, 28.0640	Urban residential area with roadside vendors and open food display
MTN Taxi Rank	Johannesburg	2	30	–26.1995, 28.0485	Major transport hub with intense human movement and food vending

### Rationale for sample strategy applied

This study used a non-probability convenience sampling strategy because informal markets are unstructured and vendor locations and operating hours change over time. This approach enabled non-random selection of samples from high-footfall zones and increased the likelihood of identifying bacterial contaminants inherent in common market practices. Although not random, this strategy is suitable for exploring food safety research in the informal sectors that lack official registries or census data.

### Bacterial count and isolation

Bacterial isolation was conducted using nutrient agar (Sigma-Aldrich, Merck Life Science, South Africa) as culture medium. Nutrient Agar was used as a general medium and to determine the total viable bacterial counts of different microbial populations in dried fish. Each dried fish sample was weighed aseptically using a precision balance (Kern Analytic balance Z741077, South Africa), and 10 grams of each sample was aseptically transferred into 90 mL of buffered peptone water (Merck Life Science, South Africa) and homogenized using a vortex mixer. A 10-fold serial dilution was conducted from 10-1 up to 10-7 [33, 34]. From each prepared serial dilution, 1 mL was pipetted and plated in duplicate on nutrient agar using the pour plate method. Plates were then incubated at 30 ± 1 °C for 24-48 h under aerobic conditions to allow the growth and enumeration of total viable bacteria. Two consecutive dilutions with colony counts ranging from 30 to 300 Colony-forming units (CFUs)/plate were used to determine the total bacterial count (TBC). Bacterial count was calculated using this formula:

Where, N= number of CFU per gram of samples ∑C= Sum of colonies counted on all plates from two consecutive dilutions, n1= number of plates for the first dilution, n2 = number of plates for the second dilution, and d = dilution factor corresponding to the first dilution.

Isolated bacteria were selected based on morphological features and subjected to subculturing, further examination (including Gram staining), and conventional biochemical tests (including catalase, oxidase, and indole). Isolated bacteria displaying diverse biochemical profiles were preserved and subjected to molecular identification [33, 34]. In this study, bacterial isolation was first conducted using a culture-based method on nutrient agar under strict aerobic conditions. No selective anaerobic culturing method was used to target anaerobic bacteria, such as *Clostridium botulinum*. Therefore, identification of Clostridium-related isolates in this study was based solely on subsequent *16S rDNA *amplification and DNA sequencing and should be interpreted with caution.

### Phenotypic identification

A Gram stain was used to differentiate Gram-positive and Gram-negative bacteria, as described by Purkayastha *et al*. [35]. A single bacterial colony was smeared on a slide, heat-fixed, and sequentially stained with crystal violet, iodine, alcohol, and safranin. Under 100× magnification with immersion oil, Gram-positive bacteria appeared violet-blue, while Gram-negative bacteria stained pink-red. Bacterial morphology was also observed. The catalase test was conducted following Montso *et al*. [36] to identify catalase-positive bacteria, such as Enterobacteriaceae, by adding 3% hydrogen peroxide to bacterial isolates; the formation of oxygen bubbles indicated a positive result. The oxidase test, as described by Gerth *et al.* [37], involved smearing bacteria on oxidase strips; a rapid blue coloration indicated oxidase positivity. The indole test, as described by Hassan *et al. *[38], was performed using Kovac’s reagent on tryptone broth cultures; a red ring signified indole production.

### Molecular approach

The research used *16S rDNA *amplification and sequencing with phylogenetic reconstruction to identify bacterial isolates at the species level. This molecular approach is more robust than conventional phenotypic and biochemical methods, which are frequently used in similar studies, and it enables evaluation of evolutionary relationships among isolates. This type of methodology is not fully exploited in research on dried fish in African informal markets.

### Extraction and purification of genomic DNA

The extraction and purification of the gDNA (genomic DNA) from pure culture was performed using the Zymo-Research Fungal/Bacterial DNA kit (Inqaba Biotec, South Africa), following the manufacturer’s instructions. The extraction process started by inoculating a colony into nutrient broth and incubated overnight at 37˚ C, then pelleting it down before collecting supernatant to isolate genomic DNA. The Zymo-Spin III-F filter received the supernatant solution to which DNA pre-wash buffer and gDNA wash buffer were added before DNA elution buffer was applied. The DNA was then eluted in a sterile 1.5 mL microcentrifuge tube [13]. The gDNA purity and concentration were assessed using a Nanodrop spectrophotometer (Thermo Scientific, USA). The gDNA samples with A260/280 ratios ranging from 1.8 to 2.0 were considered pure DNA for this study. The integrity of the gDNA was verified by electrophoresis on 1% agarose gel stained with ethidium bromide and visualized under UV light.

### Amplification of *16S rDNA*

Bacterial identification in this study depended on polymerase chain reaction (PCR)-based amplification of the *16S rDNA *gene sequence [39]. A 50 µL reaction volume served as the basis for carrying out the amplification reaction, which contained: 25 µL (PCR Master Mix), 2 µL (DNA template), 19 µL (nuclease-free water), 4 µL oligonucleotide primer (universal primers) 27F (5'-AGA GTT TGA TCC TGG CTC AG-3') and 1492R (5'-ACG GCT ACC TTG TTA CGA CTT 3'), at a concentration of 25 µM. These primers were synthesized by Inqaba Biotechnical Industrial (Pty) Ltd, in Pretoria, South Africa. All these reagents were mixed in the PCR tubes. The PCR tubes were then loaded into the PCR machine (Bio-Rad T100TM thermal cycler). The conditions applied for the amplification were set as follows: One initial cycle of 95°C for 30 sec, Denaturation 35 cycles at 94°C for 30 sec, annealing at 50°C for 30 sec, Extension at 72°C for 2 min, Final extension cycle of 72°C for 10 min, followed by incubation at 4° C indefinitely. Nuclease-free water (negative control) was used in all PCR reactions to monitor contamination. The expected DNA amplicon size was approximately 1,465bp.

### Agarose gel electrophoresis

A 1% agarose gel was used to separate the amplified PCR products. The following is how the agarose gel was made: After weighing and combining 1g of agarose gel with 100mL of Tris-acetate-EDTA buffer, the agarose was microwave-dissolved for five min. The gel was then allowed to cool at roughly 40°C, and 0.5 mL of ethidium bromide was added for staining. After casting, the gel was left to solidify. Following the gel's placement within the electrophoresis chamber, 5 μL of DNA and 5 μL of loading dye were combined and moved to one of the gel electrophoresis tank's walls. The 100 bp DNA ladder was employed. The electrophoresis was run for forty-five minutes at 80 volts and 400 MA. The Gel Doc imaging equipment (Bio-Rad Chemi Doc^TM^) was then used to visualize the gel. Successful amplification was indicated by a single, distinct band (DNA fragment) in each sample. Image Lab (version 6.00.22) software was used to capture the DNA bands. After electrophoresis, the PCR products were delivered to Inqaba Biotechnical Industrial (Pty) Ltd, Pretoria, South Africa, for sequencing.

### DNA sequencing

The amplified *16S rDNA *products were sent to Inqaba Biotechnologies in Pretoria, South Africa, for sequencing. Sanger sequencing was used to purify and sequence the amplified PCR product. Finch TV (version 1.4.0) was used to inspect the raw sequence data, and low-quality regions were removed. Sequence editing and alignment were performed using BioEdit software. Furthermore, isolates were identified using a consensus sequence and BLAST (Basic Local Alignment Search Tool) in the National Center for Biotechnology Information.

### Phylogenetic reconstruction

The nucleotide BLAST analysis showed sequence similarities ranging from 95% to 100%. The neighbor-joining method was used to reconstruct the evolutionary history and phylogenetic tree [40]. The optimal tree had a total branch length of 14.33746207. The percentage of replicate trees in which the associated taxa clustered together was determined using 1,000 bootstrap replicates, with evolutionary distances calculated using the Kimura 2-parameter model [41]. Both the scaled tree branches and the network of evolutionary distance data used in phylogenetic reconstruction were scaled in the depicted visual. The evolutionary distances were determined by the number of base differences per site by the p-distance method.[42]. This analysis included 48 nucleotide sequences. First-, second-, third-, and noncoding codon locations were addressed. For any pair of sequences, all ambiguous places were removed (pairwise deletion option). The final dataset consisted of 1552 locations in total. In MEGA X, phylogenetic analyses were performed [43]. After depositinion to NCBI, the isolates’ full *16S rDNA *gene sequences were assigned accession numbers.

### Antibacterial susceptibility test

Antibiotic susceptibility was determined using the disk diffusion method [44]. Antimicrobial susceptibility testing was performed on purified and identified isolates with *16S rDNA *and DNA sequencing. An individual colony was inoculated into 5 mL of nutrient broth and incubated overnight. Then, 1 mL of inoculum was transferred, spread evenly across the entire surface of Mueller-Hinton agar (Sigma-Aldrich, South Africa), and dried for 1 min. Antibiotic discs at different concentrations (Table 2) were added to Mueller-Hinton agar and incubated at 37 °C for 24 h. As soon as the incubation period ended, the measurement of the zone of inhibition for each inoculated site using a meter rule was taken and the result was recorded. We analyzed the results using the standards produced by the Clinical Laboratory Standards Institute (Table 2). The evaluation of the method was made with the use of standardized quality test organisms: *S. aureus *American Type Culture Collection (ATCC®) 29213 and *E. coli* ATCC® 25922 [45]. Breakpoints were applied based on bacterial grouping, where Gram-positive organisms (e.g., *Staphylococcus* spp. and *Enterococcus* spp.) and Gram-negative organisms (e.g., *Klebsiella* spp. and *Enterobacter *spp.) were interpreted using the corresponding Clinical and Laboratory Standards Institute (CLSI) criteria. The antibiotics selected in this study were chosen based on their availability and relevance to commonly used antimicrobial agents in both human and veterinary practice in South Africa. Antimicrobial susceptibility testing quality control was performed using *S. aureus* ATCC 29213 and *E. coli* ATCC 25922, which were tested with every batch of isolates. The inhibition zone diameters fell within the acceptable ranges suggested by CLSI (2020), validating the results.

**Table 2:** Guideline of antibiotic resistance according to the Clinical Laboratory Institute (CLSI, 2020).

**Antibiotics**	**Abbreviation**	**Dis** **k** ** content (µg)**	**Applicable bacterial group**	**Susceptible (S) (mm)**	**Intermediate (I) (mm)**	**Resistant (R) (mm)**
Amoxicillin	AML10	10 µg	Gram-positive and Gram-negative	≥ 18	14–17	≤ 13
Gentamicin	CN10	10 µg	Gram-negative (Enterobacteriaceae)	≥ 15	13–14	≤ 12
Norfloxacin	NOR5	5 µg	Gram-negative (Enterobacteriaceae)	≥ 17	13–16	≤ 12
Ciprofloxacin	CIP5	5 µg	Gram-positive and Gram-negative	≥ 26	22–25	≤ 21
Chloramphenicol	C30	30 µg	Gram-positive and Gram-negative	≥ 18	13–17	≤ 12
Erythromycin	E15	15 µg	Gram-positive (Staphylococcus spp., Enterococcus spp.)	≥ 23	14–22	≤ 13
Streptomycin	S10	10 µg	Gram-positive and Gram-negative	≥ 25	18–24	≤ 17

AML = Amoxicillin; C = Chloramphenicol; CIP = Ciprofloxacin; CN = Gentamicin; E = Erythromycin; NOR = Norfloxacin; S = Streptomycin.

### Statistical analysis

The statistical analyses were performed using IBM SPSS Statistics version 27.0. Descriptive statistics that were used to summarize the data included means, standard deviations, frequencies, and percentages. All microbiological count results were log₁₀-transformed (log_10_ CFU/g) before statistical analysis to improve variance homogeneity and facilitate comparison among samples. Analysis of the transformed data was performed using statistical methods in Microsoft Excel. The Shapiro-Wilk test was used to determine whether the transformed data were normally distributed. Where the conditions of normality and homogeneity of variance were satisfied, a General Linear Model was used with a fixed-effect model to calculate the least-squares means [22]. The differences among groups were analyzed by one-way analysis of variance (ANOVA) and then post hoc Bonferroni comparisons were made with the significance level of p < 0.05. An alternative nonparametric test, the Kruskal-Wallis test, was used when the parametric assumptions were not satisfied.

## RESULTS

### Worksheet data from the informal markets

**Figure 1:**
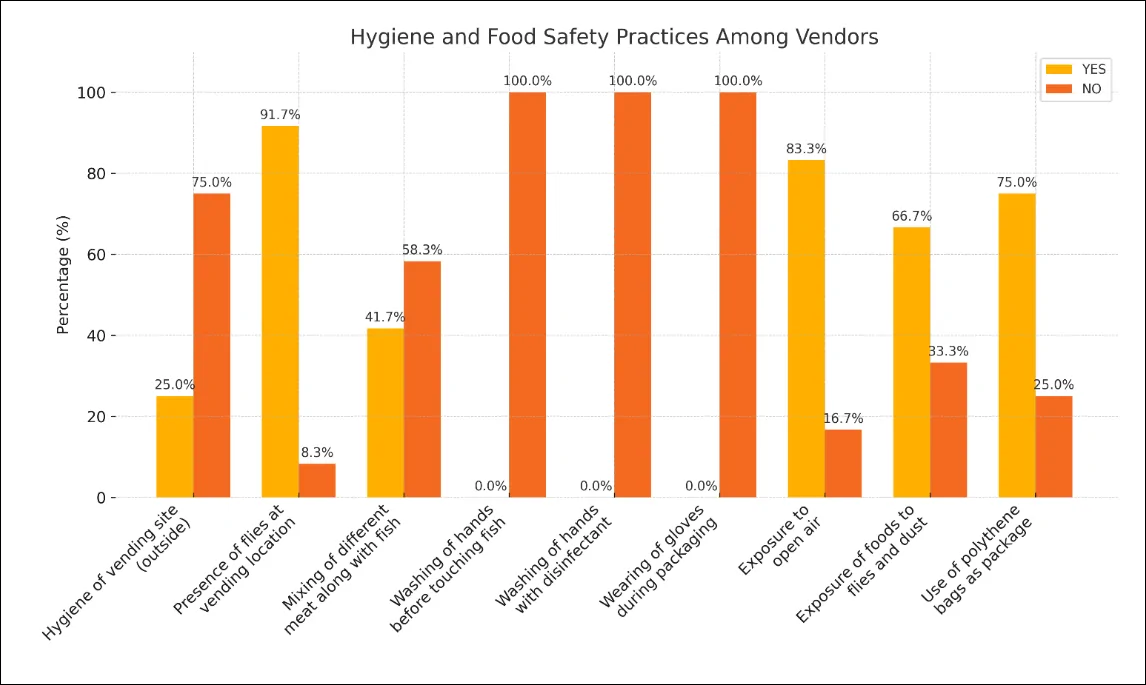
Representative hygienic conditions observed at informal dried fish vending sites in Gauteng Province, South Africa. The images illustrate poor sanitary conditions, including flies, stagnant water, and a lack of appropriate personal protective equipment among vendors. These unhygienic practices may increase the risk of microbial contamination and compromise the microbiological safety of dried fish sold in informal markets. Data are presented as mean ± standard error (SE).

All observations included 12 food sellers, as indicated in the observation checklist (Table 3). Most vendors (58.3%; 7/12) were between 30 and 49 years old. Notably, a quarter (n = 3/12) of participants were aged 29. Alternatively, just 16.6% (n = 2/12) of the sample were over 50. According to Table 3, women accounted for more than two-thirds of all food vendors (66.6%). The sample consisted of 8 females (n = 8/12) and 4 males (n = 4/12). Regarding biosecurity at the selling points, this investigation found flies at 66.6% (n = 8/12) of the vending locations. Additionally, 100% monitoring across all selling points (n = 12/12) showed that food handlers/sellers were not using personal protective equipment or following decontamination procedures for food safety. Furthermore, Figure 1 lists additional unsanitary behaviors identified during this investigation.

**Table 3:** Demographic information of the participants and their characteristics.

**Variables**	**Number of participants (n)**	**Percentage**
Age of Participants		
<29 years	3	25
30 to 49	7	58.3
>50	2	16.6
Gender		
Females	8	66.6
Males	4	33.3

Results from the checklist-based observation during sample collection showed that 25% (n = 3/12) of markets were arranged neatly and in order on the street and inside the shop. Seventy-five percent (n = 9/12) of the streets were filled with stagnant water with an unpleasant smell and dirt, and the waste was dumped opposite or closer to the markets. In all vending places, 100% (n = 12/12) of vendors were observed not applying hygienic measures. Among the samples collected, none were stored in the fridge; we assume that, since the fish was dried, the sellers didn’t worry much about spoilage. In addition, 75% (n = 9/12) of vendors were observed using plastic bags to pack their product. Moreover, 66.7% (n = 8/12) of the vending places were infested with flies and other insects, and some dried fish were spoiled, although not yet sold to consumers. When collecting dried fish, 83.3% (n = 10/12) were exposed to an open area even though some were packaged in plastic.

### Descriptive summary of TBC

**Figure 2:**
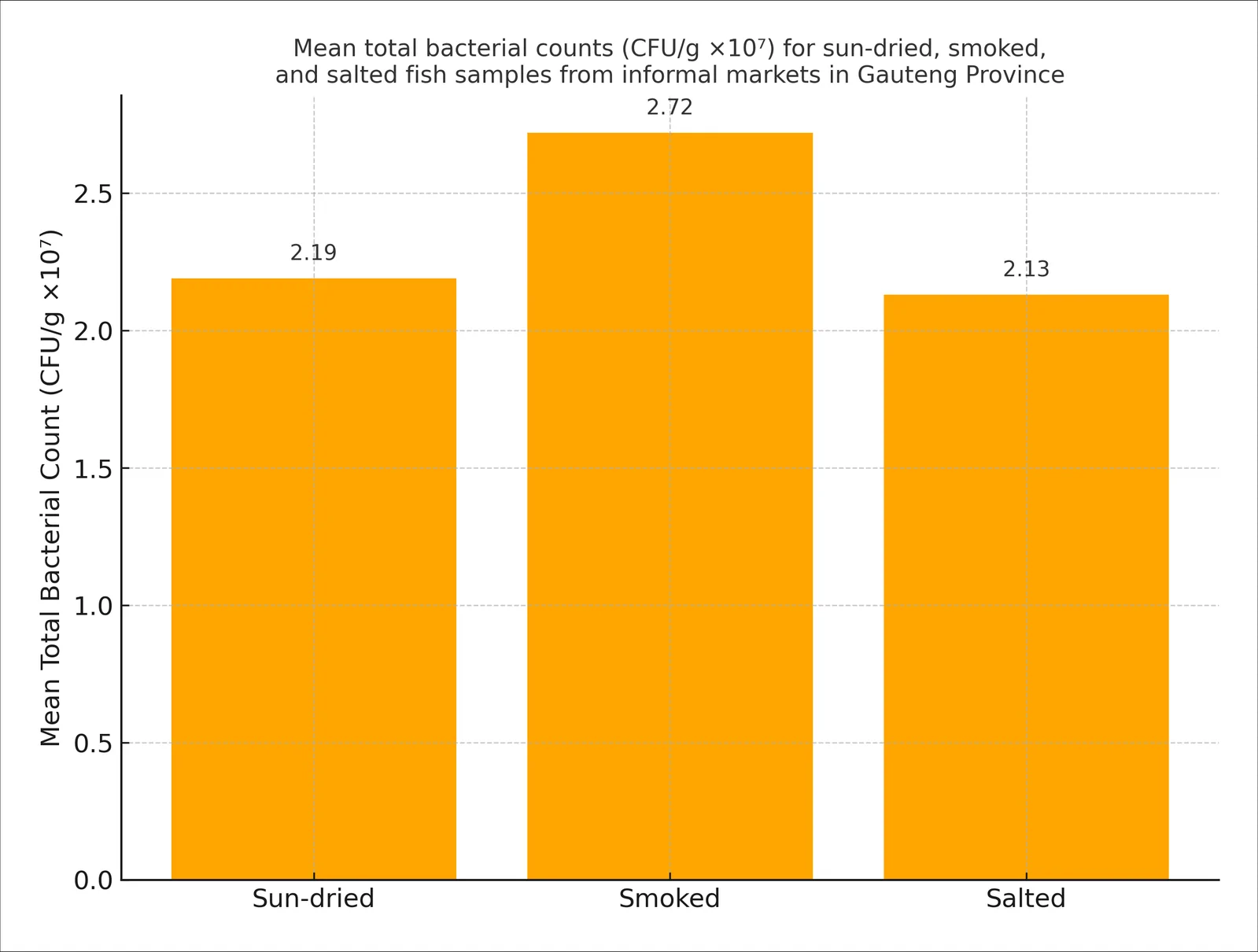
Total bacterial counts in sun-dried, smoked, and salted fish collected from informal markets in Gauteng Province, South Africa. Sun-dried fish exhibited the highest bacterial load, followed by smoked fish, whereas salted fish showed the lowest bacterial counts. The higher bacterial contamination observed in sun-dried fish is likely attributable to prolonged exposure to environmental contaminants during drying, handling, storage, and marketing. Data are presented as mean ± standard error (SE).

**Statistical results for TBC:** TBC varied among the three types of dried fish. Salted fish showed counts ranging from 0.8 × 10⁷ to 3.2 × 10⁷ CFU/g, while sun-dried fish ranged from 1.0 × 10⁷ to 4.8 × 10⁷ CFU/g. Smoked fish exhibited counts ranging from 1.0 × 10⁷ to 5.34 × 10⁷ CFU/g. The mean bacterial counts were 2.13 × 10⁷ CFU/g for salted fish, 2.91 × 10⁷ CFU/g for sun-dried fish, and 2.72 × 10⁷ CFU/g for smoked fish. These results indicate variability in microbial contamination across fish types, as shown in Figure 2.

**Comparison of TBC among different fish species:** The results suggest that differences in bacterial counts among fish species are statistically significant at the 5% level (p < 0.05). This result shows that bacterial counts varied by fish type.

Results show that differences in average bacterial counts in the fish are statistically significant at the 5% level. Findings from a test of among-subject variation further suggest that bacterial counts are influenced by fish type. The raw mean values of TBC of the three fish types (sun-dried, smoked and salting) were 2.91 × 10⁷ CFU/g, 2.72 × 10⁷ CFU/g and 2.13 × 10⁷ CFU/g, respectively. The 95% confidence intervals (CI) for the means of bacterial loads were as shown below: sun-dried fish (2.45 × 10⁷–3.36 × 10⁷ CFU/g), smoked fish (2.26 × 10⁷–3.18 × 10⁷ CFU/g), and salted fish (1.68 × 10⁷ CFU/g). These intervals were determined from the transformed (log₁₀ CFU/g) data to normalize the distribution and ensure valid statistical inference. To offset any potential Type I errors based on multiple pairwise comparisons across fish types, Bonferroni correction was adopted on the set of post hoc tests. The p-value level, p < 0.017 (0.05/3 comparisons), following the Bonferroni correction (adjusted α = 0.017), pairwise t-test revealed that the difference between the sun-dried and salted fish could also be considered significant (adjusted p = 0.012). However, the statistical significance was not achieved after adjustment between sun-dried and smoked fish (p = 0.058) and smoked and salted fish (p = 0.041). The results suggest that differences in bacterial counts are mainly attributable to differences between sun-dried and salted fish.

### Bacterial counts from the four areas

**Figure 3:**
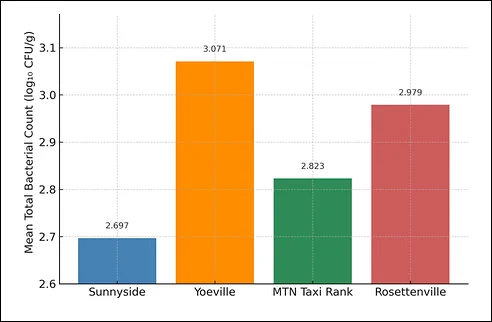
Total bacterial counts in dried fish collected from different informal market locations in Gauteng Province, South Africa. No significant differences in bacterial counts were observed among sampling locations (p > 0.05), indicating comparable microbial contamination levels across the surveyed markets. These findings suggest that similar handling, storage, and hygiene practices may be prevalent throughout the informal market system in the study area. Data are presented as mean ± standard error (SE).

No meaningful difference (p > 0.05) was found when comparing the areas in Figure 3using sample means from the different vending locations. Overall, the mean values were similar. The average range of Sunnyside samples was lower than that of Yeoville samples; however, the difference was not statistically significant (p > 0.05).

The results show that the differences in the mean bacterial counts across the four areas are not statistically significant at the 5% level (p > 0.05). This implies that the mean bacterial count does not depend on area. This was confirmed by a test of between-subject effects and post hoc.

### Bacterial counts’ distribution across the study areas

The between-subjects effects test assessed whether area was significantly related to the number of bacteria (n × 10⁷). Table 4 shows no significant difference in the mean number of bacteria across areas (p > 0.05).

**Table 4:** Distribution of bacterial counts between the areas.

**Source**	**Type III Sum of Squares**	**df**	**Mean Square**	**F**	**Significance**
Corrected Model	3.136^a^	3	1.045	0.873	0.457
Intercept	1115.582	1	1115.582	931.372	0.000
Area	3.136	3	1.045	0.873	0.457
Error	162.898	136	1.198		
Total	1314.865	140			
Corrected Total	166.035	139			

^a.^ R Squared = 0.019 [Adjusted R Squared = -0.003].

### Tests of between-subjects effects dependent variable: TBC (n × 10⁷)

There is no statistically significant difference among the four locations, as indicated by the p-value for the mean bacterial count (p > 0.05). Accordingly, the findings show that the bacterial count is independent of the sampled vending location.

**Bacterial identification and confirmatory results based on molecular analysis using the** *16S rDNA* **test result for bacterial confirmation**

**Figure 4:**
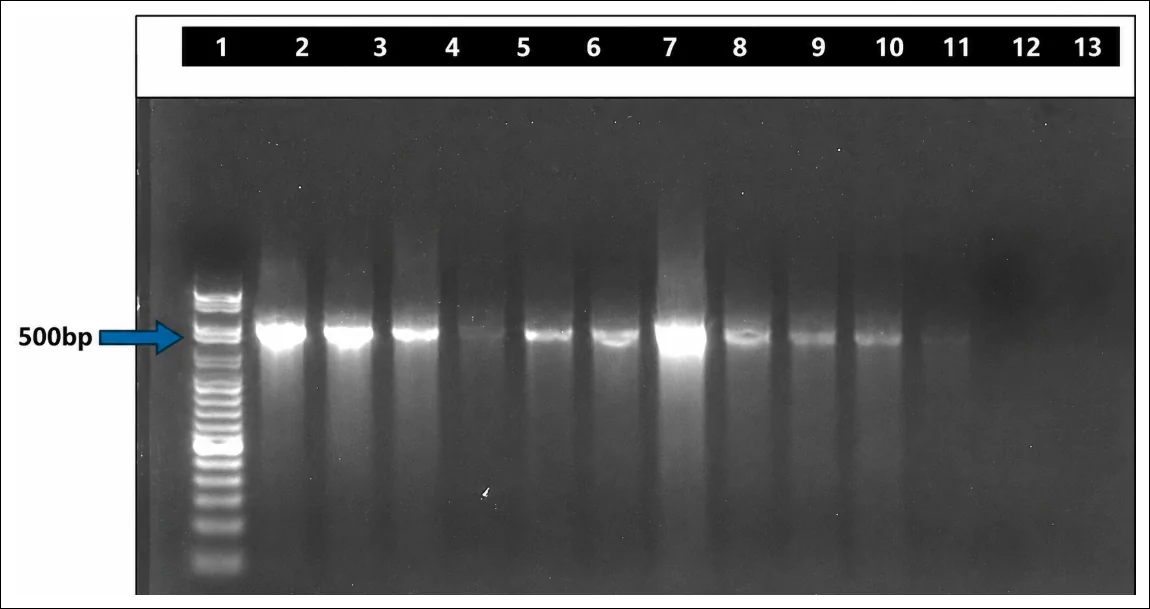
Agarose gel electrophoresis of amplified *16S rDNA* polymerase chain reaction products from representative bacterial isolates recovered from dried fish samples. From left to right: lane L, 100 bp DNA ladder; lane 1, *Staphylococcus aureus*; lane 2, *Klebsiella pneumoniae*; lane 3, *Staphylococcus xylosus*; lane 4, *Staphylococcus* spp.; lane 5, *Paraclostridium* *bifermentans*; lane 6, *Enterococcus faecalis*; lane 7, *Lysinibacillus* *macroides*; and lane 8, *Enterococcus faecalis*. Lanes 9 and 10 represent the positive controls, *Staphylococcus aureus* ATCC® 29213 and *Escherichia coli* ATCC® 25922 (JGK, Lab Africa, South Africa), respectively, whereas lanes 11 and 12 contain DNA-free water as negative controls. The presence of distinct amplicons confirms successful amplification of the bacterial *16S rDNA* gene.

Sequences showing similarity to *Clostridium* spp. were detected in 10 of 67 isolates (14.94% of the total) by *16S rDNA *and DNA sequencing analysis. The *Staphylococcus* spp. molecular confirmation test and *Staphylococcus* *xylosus* identification rates reached 11.94% (8/67) and 8.95% (6/67), respectively, as shown in Table 5. The occurrence data for other bacteria varied, as shown in Table 5 and in the gel electrophoresis in Figure 4.

**Table 5:** Confirmatory results based on 16S rDNA.

**Confirmed Organism**	**Number of Isolates**	**Percentag** **e**
*Clostridium* spp.	10	14.92
*Staphylococcus* spp.	8	11.94
*Staphylococcus * *xylosus*	6	8.95
*Klebsiella pneumoniae*	5	7.46
*Enterococcus faecalis*	4	5.97
*Staphylococcus * *lentus*	4	5.97
*Paraclostridium* *bifermentans*	4	5.97
*Klebsiella* spp.	3	4.47
*Staphylococcus aureus*	3	4.47
*Clostridium * *bifermentans*	3	4.47
*Enterococcus faecium*	2	2.98
*Clostridium botulinum*	2	2.98
*Corynebacterium * *variabile*	2	2.98
*Planococcaceae bacterium*	2	2.98
*Staphylococcus saprophyticus*	2	2.98
*Staphylococcus * *sciuri*	2	2.98
*Lysinibacillus* *macroides*	1	1.49
*Micrococcus * *caseolyticus*	1	1.49
*Micrococcus caseolyticus* subsp. *hominis*	1	1.49
*Enterobacter* spp.	1	1.49
*Enterobacter * *ludwigii*	1	1.49
Total	67	100

**Figure 5:**
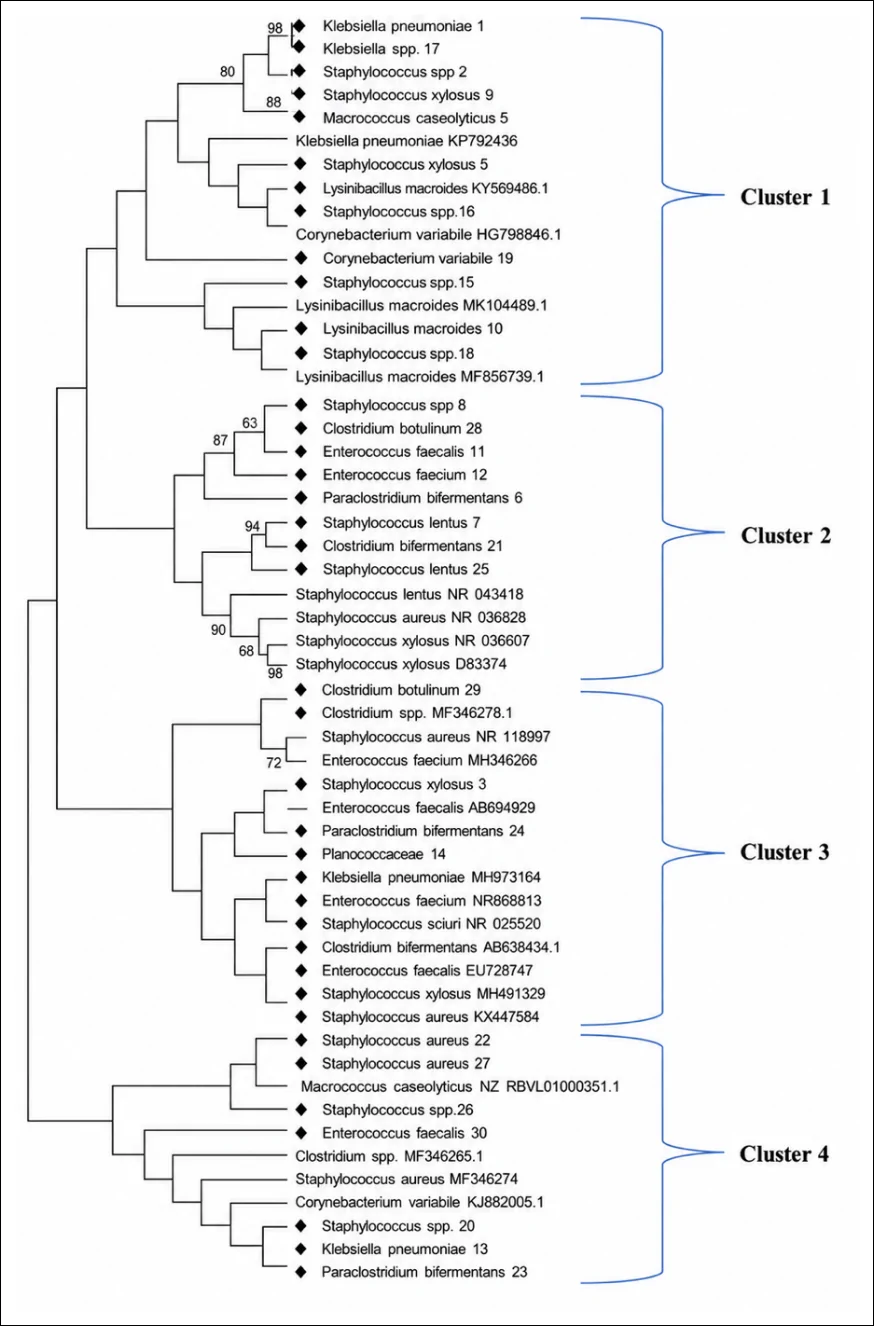
Phylogenetic tree of bacterial isolates recovered from dried fish samples based on 16S rRNA gene sequences and constructed using the neighbor-joining method. Bootstrap values derived from 1,000 replicates are shown at the branch nodes to indicate the robustness of the inferred phylogenetic relationships. Evolutionary distances were calculated using the p-distance method. Isolates generated in the present study are identified by the symbol (♦), whereas the remaining sequences represent the closest reference strains retrieved from the GenBank database.

**Bacterial similarities and their accession numbers:** Table 6 presents the bacterial species identified in this study, along with their corresponding GenBank accession numbers. These sequences were subsequently used for phylogenetic analysis to determine the evolutionary relationships among the isolates, as illustrated in Figure 5.

### Distribution of important food pathogens recovered from different types of fish samples

**Figure 6:**
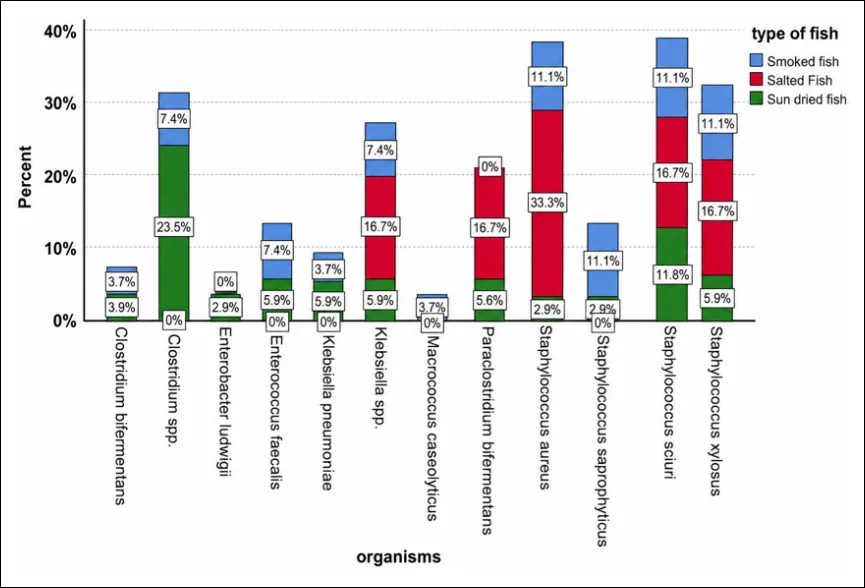
Distribution of identified bacterial species according to fish processing methods (sun-dried, smoked, and salted) in informal markets of Gauteng Province.

**Figure 7:**
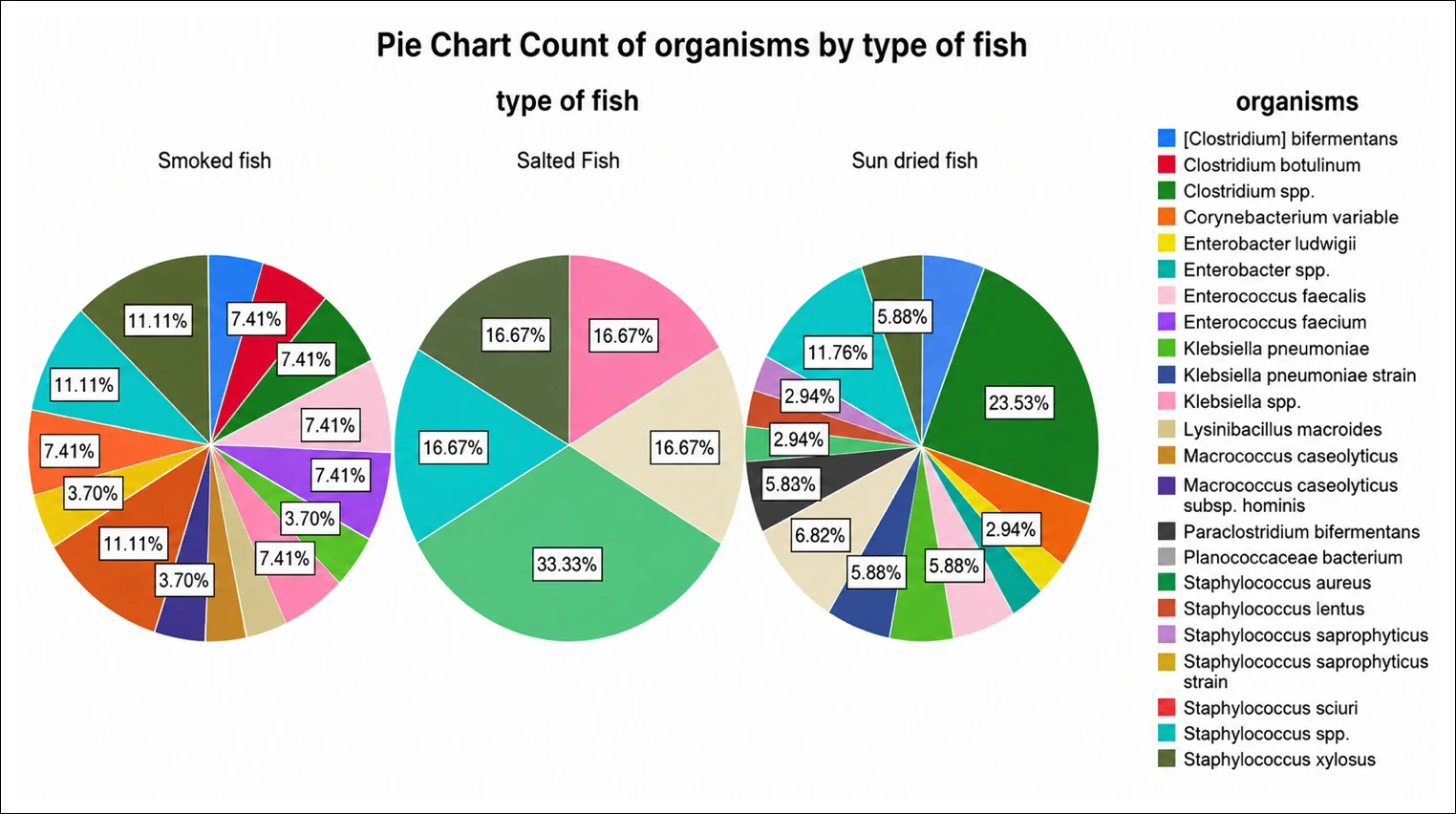
Distribution of bacterial species identified in dried fish samples based on *16S rDNA* gene sequencing. *Staphylococcus* spp., *Klebsiella* spp., and *Clostridium* spp. were the most frequently detected bacterial groups across all fish types, indicating their predominance among the bacterial isolates recovered in this study.

Figures 6 and 7 show the percentages of the principal bacteria isolated in this investigation. The results showed the types of dried fish and the proportion of each organism present. Only sun-dried fish samples had 100% of the following pathogens: Planococcaceae bacteria, *Corynebacterium variabile*, *Enterobacter* spp., and *Enterobacter* *ludwigii*, *Enterococcus faecium*, *Klebsiella* species, *Lysinibacillus* *macroides*, *Macrococcus* *caseolyticus, Micrococcus* *caseolyticus* subsp. *hominis*, and *Staphylococcus* *sciuri* were all 100% present in the smoked fish. Additionally, *Staphylococcus* spp. infection in the salted fish.

**Table 6:** 16S rDNA sequences and their accession number.

**Sequence_ID**	**Reference from NCBI database**	**Accession no. from the GenBank**	**Obtained Accession no.**	**S** **imilarity (%)**
Seq1	*Klebsiella pneumoniae*	MH973164	MW078395	99
Seq2	*Staphylococcus* spp.	KT151895	MW078396	99
Seq3	*Staphylococcus * *xylosus*	MK253321	MW078397	100
Seq4	*Staphylococcus * *xylosus*	KC456590	MW078398	99
Seq5	*Macrococcus* *caseolyticus*	NR_159094	MW078399	99
Seq6	*Paraclostridium* *bifermentans*	MK894870	MW078400	100
Seq7	*Staphylococcus * *lentus*	MF678888	MW078401	98
Seq8	*Staphylococcus* spp.	HM584794	MW078402	99
Seq9	*Staphylococcus * *xylosus*	JX035942	MW078403	99
Seq10	*Lysinibacillus* *macroides*	MG892813	MW078404	100
Seq11	*Enterococcus faecalis*	MK254994	MW078405	99
Seq12	*Enterococcus faecium*	MK748256	MW078406	99
Seq13	*Klebsiella pneumoniae*	CP040363	MW078407	97
Seq14	Planococcaceae	LK934680	MW078408	99
Seq15	*Staphylococcus* spp.	KU245713	MW078409	99
Seq16	*Staphylococcus* spp.	KU644384	MW078410	98
Seq17	*Klebsiella* spp.	KJ143756	MW078411	99
Seq18	*Staphylococcus* spp.	JX944828	MW078412	98
Seq19	*Corynebacterium * *variabile*	KP140842	MW078413	99
Seq20	*Staphylococcus* spp.	KJ504153	MW078414	99
Seq21	*Clostridium * *bifermentans*	KP944171	MW078415	99
Seq22	*Staphylococcus aureus*	MK780044	MW078416	97
Seq23	*Paraclostridium* *bifermentans*	MH346281	MW078417	99
Seq24	*Paraclostridium* *bifermentans*	MK606081	MW078418	99
Seq25	*Staphylococcus * *lentus*	MK439492	MW078419	99
Seq26	*Staphylococcus* spp.	KC688883	MW078420	99
Seq27	*Staphylococcus aureus*	LR134268	MW078421	99
Seq28	*Clostridium botulinum*	CP028859	MW078422	97
Seq29	*Clostridium botulinum*	CP013243	MW078423	99
Seq30	*Enterococcus faecalis*	MH250054	MW078424	99

*Staphylococcus* spp. were more commonly detected on the salted fish compared to the other types of fish. All the dried fish types were mainly contaminated with *Klebsiella* spp., *Staphylococcus* spp., and salted and smoked fish were also highly contaminated with *Clostridium* spp.

The prevalence of organisms and the type of dried fish do not statistically significantly correlate, according to the chi-square test of association (Table 7) (p > 0.05).

### Antimicrobial susceptibility results

**Figure 8:**
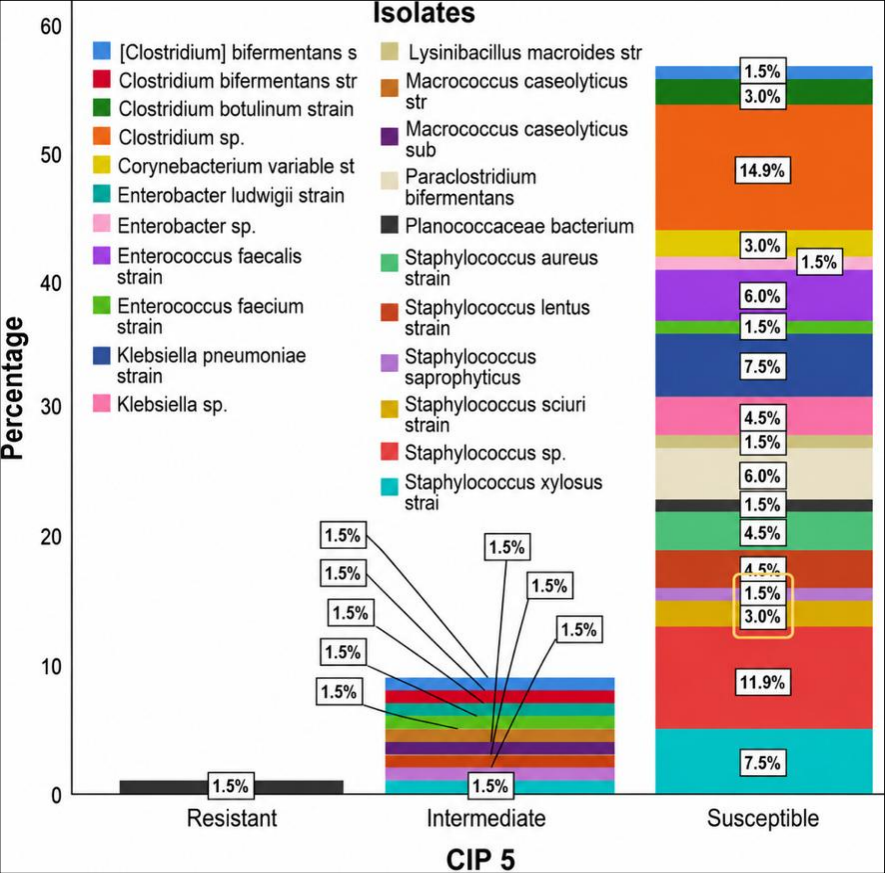
Ciprofloxacin (CIP 5) resistance profile of bacterial isolates recovered from dried fish samples. The majority of isolates were susceptible to ciprofloxacin, whereas resistance was observed only in *Staphylococcus aureus*. A smaller proportion of isolates exhibited intermediate susceptibility, indicating that ciprofloxacin remained effective against most bacterial species identified in this study.

**Figure 9:**
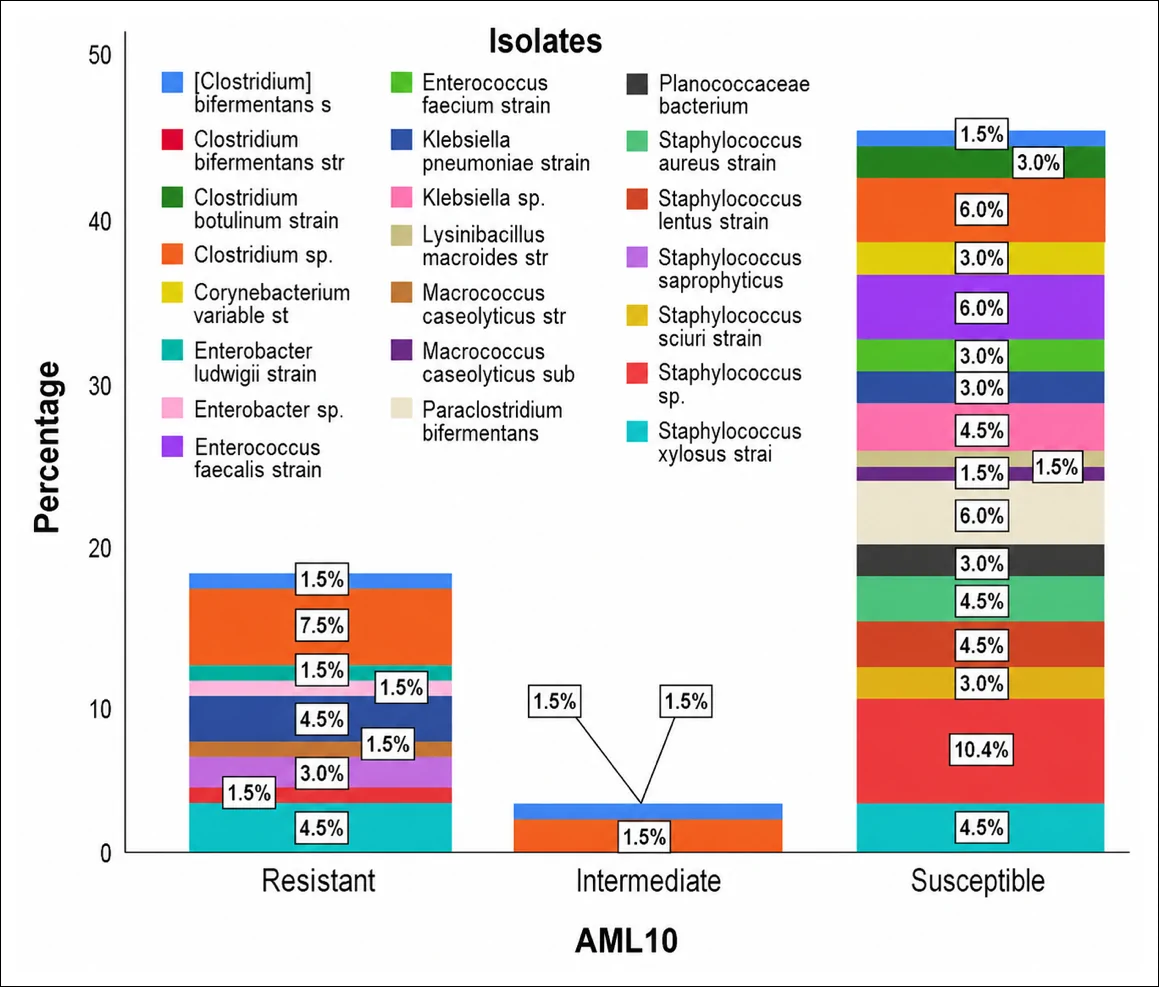
Amoxicillin (AML 10) resistance profile of bacterial isolates recovered from dried fish samples. Most isolates were susceptible to amoxicillin, whereas a substantial proportion were resistant, indicating reduced effectiveness of the antibiotic against certain bacterial species. Only a small number of isolates exhibited intermediate susceptibility. Overall, susceptible isolates constituted the largest group, followed by resistant isolates.

**Figure 10:**
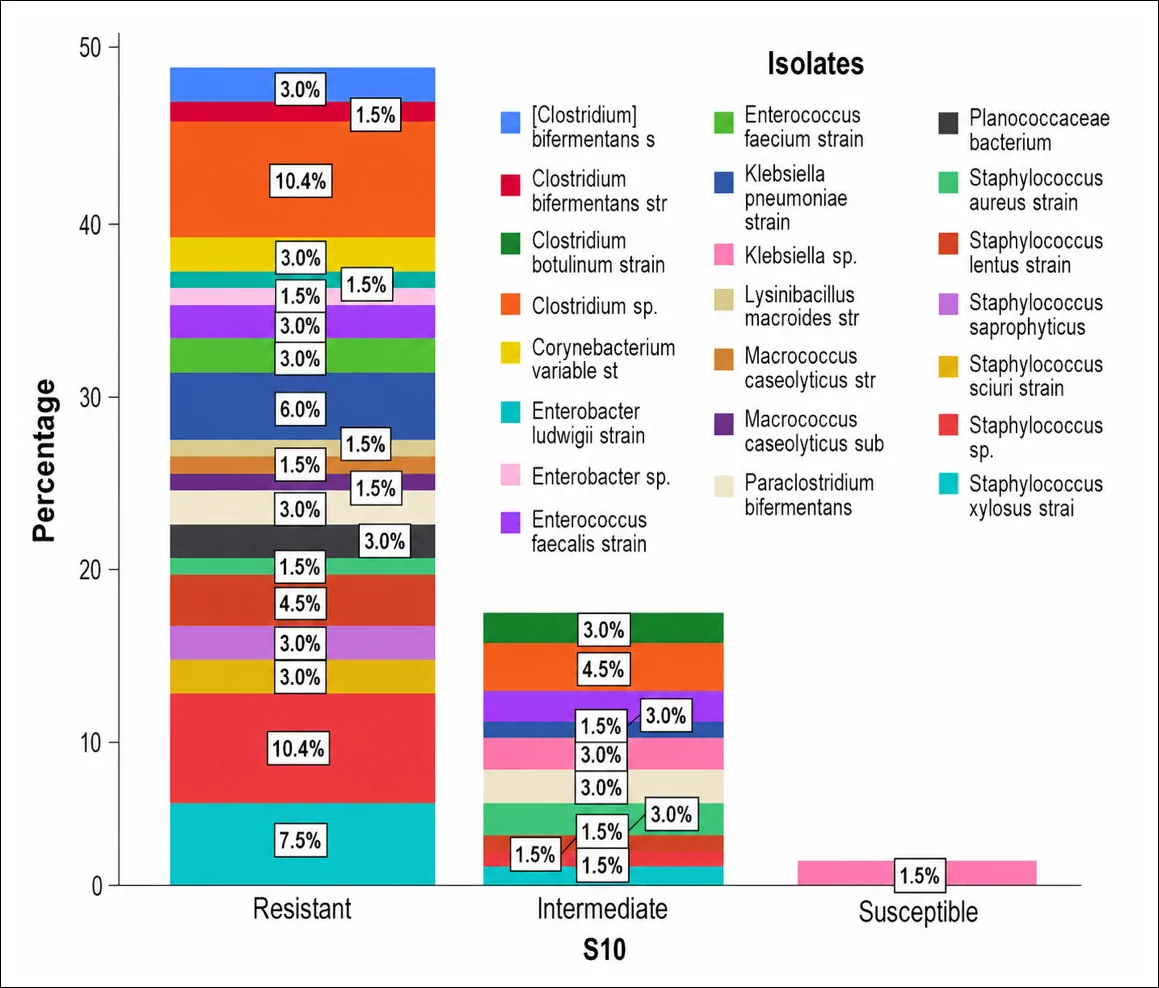
Streptomycin (S10) resistance profile of bacterial isolates recovered from dried fish samples. Most isolates were resistant to streptomycin, whereas a smaller proportion remained susceptible. Only a few isolates exhibited intermediate susceptibility, indicating that streptomycin was limited in its effectiveness against the bacterial isolates identified in this study.

**Figure 11:**
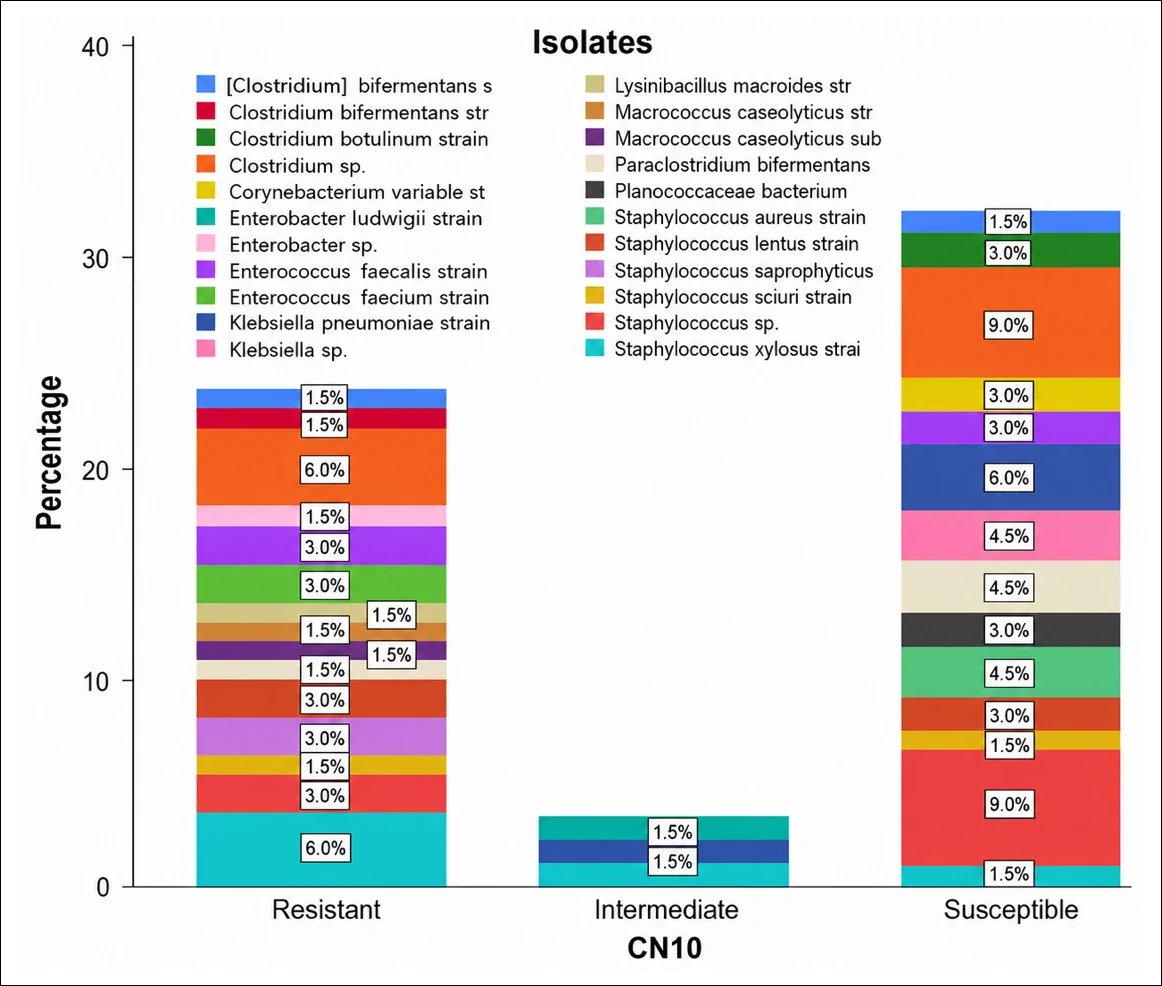
Gentamicin (CN10) resistance profile of bacterial isolates recovered from dried fish samples. Most isolates were susceptible to gentamicin, whereas resistance was observed in the second-largest proportion. Only three isolates exhibited intermediate susceptibility, indicating that gentamicin remained effective against the majority of bacterial species identified in this study.

**Figure 12:**
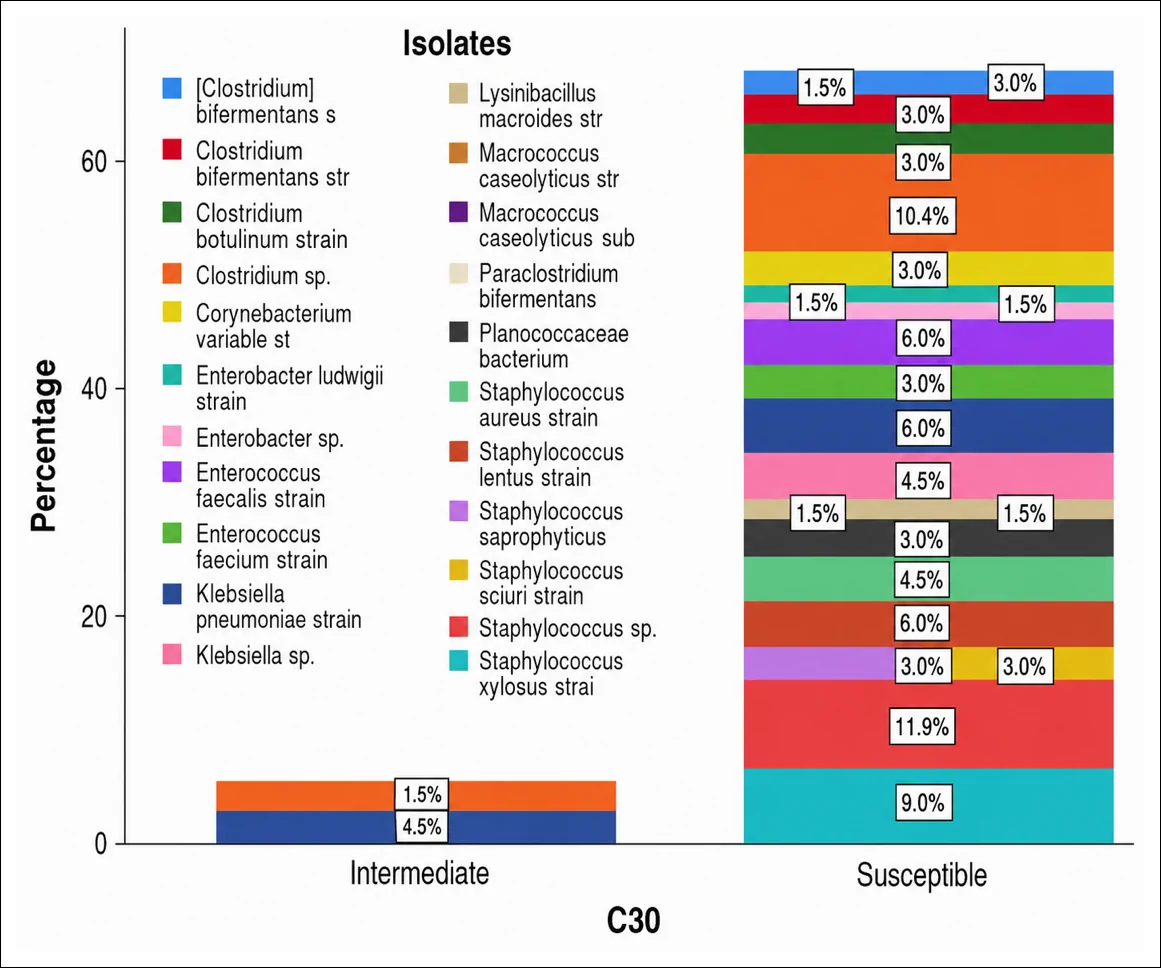
Chloramphenicol (C30) resistance profile of bacterial isolates recovered from dried fish samples. Most isolates were susceptible to chloramphenicol, whereas the remaining isolates exhibited intermediate susceptibility. No chloramphenicol-resistant isolates were detected, indicating excellent in vitro activity of this antibiotic against all bacterial species identified in this study.

**Figure 13:**
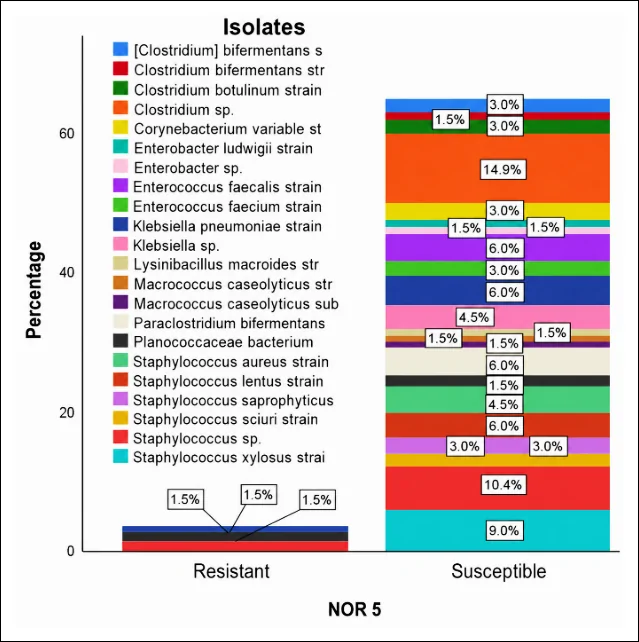
Norfloxacin (NOR5) resistance profile of bacterial isolates recovered from dried fish samples. Most isolates were susceptible to norfloxacin, whereas the second-largest proportion exhibited resistance. These findings indicate that norfloxacin retained activity against most bacterial isolates, although resistance was detected in a considerable number of strains.

**Figure 14:**
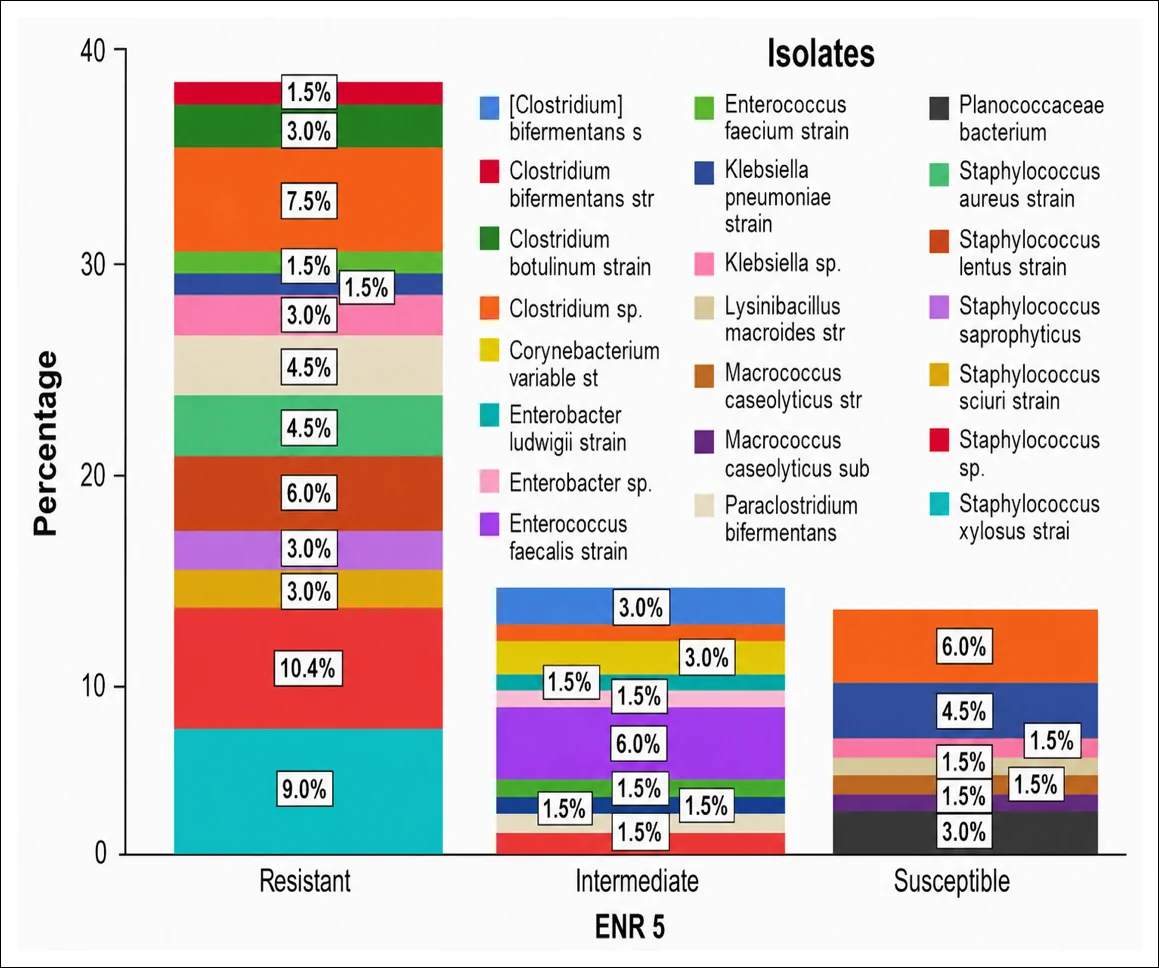
Erythromycin (E5) resistance profile of bacterial isolates recovered from dried fish samples. The majority of isolates were resistant to erythromycin, whereas the second-largest proportion exhibited intermediate susceptibility. Only a small number of isolates were susceptible, indicating limited effectiveness of erythromycin against the bacterial species identified in this study.

The AMR patterns of the bacterial isolates are shown in Figures 8–14, with different patterns of susceptibility to the different antibiotics tested. As shown in Figure 8, most isolates were susceptible to ciprofloxacin, with little resistance. Figure 9 shows that amoxicillin exhibited an intermediate resistance profile across several isolates, indicating low efficacy. Figure 10 indicates that streptomycin exhibited the highest levels of resistance, demon-strating its poor effectiveness in treating the recovered bacteria. Figure 11shows an intermediate resistance trend for gentamicin, with a percentage of isolates still susceptible. Figure 12 indicates that chloramphenicol showed high susceptibility, with most isolates indicating effectiveness. Figure 13shows that erythromycin resistance is high, indicating widespread reduced susceptibility. Lastly, Figure 14 indicates that the majority were susceptible to norfloxacin, and only a few were resistant. Overall, these results indicate uneven resistance patterns among the identified bacterial species and suggest the presence of antimicrobial-resistant bacteria in the informal dried fish value chain, which may pose a public health concern (supplementary material S1).

Table 8 shows the association between antibiotic use and AMR among the bacterial isolates. A statistically significant correlation was observed at the 5% significance level (p < 0.05), indicating a significant relationship between antibiotic exposure and the development of resistance in the isolated bacteria.

The statistical results indicated that more than 50% of the isolated bacteria tested in this study exhibited resistance to erythromycin (E5) and streptomycin (S10). An overall resistance profile showed that 73.1% of the isolated bacteria were resistant to streptomycin and 58.2% to erythromycin. However, the overall results for all tested isolated bacteria showed that 60.1% of the isolates displayed susceptibility profiles to other antibiotic agents tested against them, as shown in Table 9.

**Table 7:** The chi-square test of association and percentage of isolated organism.

**Chi-Square Tests**	**Value**	**df**	**Asymp** **totic** **s** **ig** **nificance** ** (2-sided)**
Likelihood Ratio	52.650	40	0.087
N of Valid Cases	67		

**Table 8:** Association between resistance and antibiotic.

**Chi-Square Tests**	**Value**	**df**	**p-value**
Pearson Chi-Square	244.619^a^	12	0.000

**Table 9:** Distribution of antibiotic susceptibility patterns.

**Antibiotic**	**Intermediate**	**Resistant**	**Susceptible**
AML10	4.5%	26.9%	68.7%
C30	6.0%	0.0%	94.0%
CIP 5	13.4%	1.5%	85.1%
CN10	4.5%	38.8%	56.7%
E 5	22.4%	58.2%	19.4%
NOR 5	0.0%	4.5%	95.5%
S10	25.4%	73.1%	1.5%
Total	10.9%	29.0%	60.1%

AML = Amoxicillin; C = Chloramphenicol; CIP = Ciprofloxacin; CN = Gentamicin; E = Erythromycin; NOR = Norfloxacin; S = Streptomycin.

We analyzed the test results using the nonparametric Kruskal-Wallis test to determine whether the degree of resistance to each antibiotic differed significantly. For all antibiotics across all resistance categories, the p-value was less than 0.05. The dose of antibiotics depends largely on how much resistance develops. The post hoc test was examined to ensure that these changes are present and in the expected trend (Supplementary material S2-S4).

**Multidrug resistance profile of the isolates recovered from dried fish:** Table 10 presents the multidrug resistance (MDR) profile of the bacterial species in the dried fish samples. Some of them were found to be resistant to three or more antibiotic classes, such as *Clostridium* spp., *Enterobacter* spp., *E. faecium*, *Klebsiella pneumoniae*, *Macrococcus* *caseolyticus*, *Paraclostridium* *bifermentans*, *Planococcaceae bacterium*, and various species of *Staphylococcus*. It is worth mentioning that the *Staphylococcus* spp. (*S. xylosus* and *Staphylococcus saprophyticus*) were resistant to as many as 4 antibiotics. Conversely, other isolates such as *C. botulinum*, *C. variabile*, and *Klebsiella* spp. were less resistant. On the whole, the detection of MDR bacteria in dried fish reveals a serious food safety issue and suggests the role of informal markets in the spread of antimicrobial-resistant pathogens.

**Table 10:** Multidrug resistance profile of the isolated bacteria from dried fish.

**Bacterial species**	**No. of antibiotics with resistance**	**MDR (≥3 classes)**	**Key resistant antibiotics**
*Clostridium * *bifermentans*	4	Yes	E, AML, S, CN
*Clostridium botulinum*	1	No	E
*Clostridium* spp.	4	Yes	E, AML, S, CN
*Corynebacterium * *variabile*	1	No	S
*Enterobacter * *ludwigii*	2	No	AML, S
*Enterobacter* spp.	3	Yes	AML, S, CN
*Enterococcus faecalis*	2	No	S, CN
*Enterococcus faecium*	3	Yes	E, S, CN
*Klebsiella pneumoniae*	4	Yes	E, AML, S, NOR
*Klebsiella* spp.	1	No	E
*Lysinibacillus* *macroides*	2	No	S, CN
*Macrococcus* *caseolyticus*	3	Yes	AML, S, CN
*Macrococcus* *caseolyticus* subsp. *hominis*	2	No	S, CN
*Paraclostridium* *bifermentans*	3	Yes	E, S, CN
Planococcaceae bacterium	3	Yes	CIP, S, NOR
*Staphylococcus aureus*	2	No	E, S
*Staphylococcus * *lentus*	3	Yes	E, S, CN
*Staphylococcus saprophyticus*	4	Yes	E, AML, S, CN
*Staphylococcus * *sciuri*	3	Yes	E, S, CN
*Staphylococcus* spp.	4	Yes	E, AML, S, CN
*Staphylococcus * *xylosus*	4	Yes	E, AML, S, CN

## DISCUSSION

### Demographic characteristics and food safety practices of street vendors

One of the objectives of this study was to assess the food safety measures and practices applied by street vendors selling dried fish. The observational study results revealed several critical concerns. It was observed that 66.6% of vendors were female; this finding aligns with previous findings [46], which reported that most street vendors in Polokwane, South Africa, were young women under 40 years old. This demographic detail is noteworthy, although the study did not establish a direct link between gender and food safety practices. The study revealed that 58.3% of street vendors were between 30 and 49 years old. A study by Marutha *et al*. [46] found that experience in handling food is very important. It showed that vendors with more experience are more likely to follow safe food-handling practices. This suggests that greater experience in food handling may contribute to better adherence to safe food-handling practices. Additionally, Marutha *et al*. [46] noted in their study that most food handlers were from foreign countries, as inferred from their accents, attire, and the nature of the food sold, but the author concluded that identifying the exact country of origin was difficult without proper interviews.

The observations conducted in this study highlighted that many street vendors operated in potentially unsuitable environments, such as open areas with heavy pedestrian traffic, increasing the risk of food contamination. Different studies conducted on food hygiene and safety have reported that more than 65% of street vendors mostly operate in open-air settings, lacking proper facilities, which negatively affects food hygiene and safety [13, 46]. Furthermore, the lack of access to running water or adequate handwashing facilities, observed among 45% of vendors reporting such deficiencies, further compromises food safety [46]. Vendors’ age and gender were noted during sample collection. However, no significant association was observed between these variables and the microbial contamination levels found in the fish samples. Although this study did not find a statistically significant association between vendor gender or age and microbial contamination levels, these demographic characteristics are still important from a public health perspective. They can play an important role in hygiene education, how well training is delivered, and how interventions are developed. Although no direct association was found in the present study, it is still worth considering the age and gender of the vendors, when planning food safety education and interventions for informal vendors.

### Environmental and personal hygiene practices

In this investigation, it was observed that dried fish were often placed on tables near dusty areas and sometimes near garbage bins, usually without any packaging, which may increase the risk of contamination. These findings are consistent with those of Tshipamba *et al. *[13] and Marutha *et al. *[46], who found that inadequate water supply and poor environmental conditions adversely affect food-handling procedures. This research identified inadequate personal hygiene practices among vendors, as none of the observed vendors washed their hands before handling food or wore gloves, hair covers, and aprons. These observations are consistent with previous studies [13, 46], which demonstrated that street food vendors had strong knowledge but inadequate safe practices, increasing the likelihood of food contamination. The researchers noted during this study that storage practices posed a problem because dried fish samples were left at room temperature, resulting in spoilage indicators such as mold growth and foul odors. It is well established that a lack of water at the vending site and a poor environment negatively affect food hygiene practices, which can contribute to food spoilage, as reported by Marutha *et al.* [46]. Observational studies found that 66.7% of samples were exposed to dirt, dust, and insects, while 91.7% of markets had visible fly infestations. This reveals that environmental hygiene was poor. This underscores the need for improved environmental controls to enhance food safety.

### Microbial contamination levels in dried fish

The investigation determined the bacterial contamination levels in dried fish products, which included all three drying methods of salting, sun-drying, and smoking that appear in informal markets. The research found that bacterial counts in these food products exceeded acceptable safety limits, rendering them unacceptable for human consumption. According to Surendran *et al*. [47], the permissible limit allows dried fish to contain 1 × 10⁵ CFU/g at 37°C temperature. Even though this threshold limit is not an established regulatory limit, it serves as a widely recognized benchmark frequently cited in food microbiology studies. In this study, the mean bacterial counts in dried fish samples far exceeded the benchmark, indicating significant microbial contamination relative to the acceptable levels reported in previous studies. Additionally, the overall bacterial counts (TBC) presented in Figures 2 and 3 in this study, which reached 10⁷ CFU/g, were well over the generally accepted microbiological standards for ready-to-eat foods. South African food safety regulations (R.692 of 1997), Codex Alimentarius, and the International Commission on Microbiological Specifications for Foods standards all suggest that acceptable aerobic plate counts for dried or ready-to-eat fish products are in the range of 10⁵–10⁶ CFU/g. The high bacterial counts observed in the current study hence point to poor hygiene and the possibility of contamination in post-processing informal markets. These results indicate a massive violation of food safety expectations and a greater risk of foodborne illness among consumers of fish products at these outlets.

### Factors influencing bacterial counts across fish types and locations

Bacterial counts of salted fish examined in this research showed that the detected levels exceeded the recommended safety threshold. The bacterial contamination detected in smoked fish aligns with previous research [48] that studied bacterial levels in smoked fish products sold in Owerri. The different fish types showed varying microbial counts, which may reflect differences in processing, storage, distribution, and handling practices. since. The handling process enables the preservation of food quality and safety throughout the period during which fish is held between harvesting and ingestion [49]. The previous research [50, 51] found that poor hygiene and handling practices during fish drying or smoking may allow microorganisms to persist or multiply in these products. The lack of standardization in smoking processes may contribute to excessive microbial contamination in smoked fish because these processes depend on uncontrolled temperature, smoke quality, and humidity [52, 53]. Total microbial contamination in sun-dried fish depends on the prevailing weather conditions during drying times. The monsoon season's high humidity prevents proper drying of dried fish, allowing them to absorb moisture again and creating conditions conducive to bacterial and fungal growth [54, 55]. The highest bacterial counts was recorded in salted fish among test samples. The preservation method comprising salt reduces the food's water activity, which effectively limits microbial growth [56]. The study results were analyzed statistically and showed that fish preservation techniques create meaningful differences in bacterial population numbers (p < 0.05), thus revealing classification effects on microbial contamination rates. Sampling locations across different markets did not show differences in bacterial numbers because contamination measurements remained stable across all areas. Dried fish products must be handled properly, as existing microbial safety standards are insufficient for informal markets.

### Bacterial identification and public health implications

In this study, various bacterial species were identified across the study areas; among these, *Staphylococcus* spp., *Enterococcus* spp., *Enterobacter* spp., *K. pneumoniae*, and *C. botulinum* were most common. Although classical foodborne pathogens such as *Salmonella* spp. and *Listeria monocytogenes* were not detected in this study, opportunistic and antimicrobial-resistant bacteria remain a significant concern, especially in environments with a high proportion of immunocompromised patients. Opportunistic pathogens have the potential to contribute significantly to the burden of foodborne disease in such populations. One limitation of the study is that no selective anaerobic culture technique was applied to target *C. botulinum*. Because nutrient agar under aerobic conditions is not the most effective method for isolating obligate anaerobes, detection of *C. botulinum* using the general culture workflow and Sanger identification should be approached with caution. Selective enrichment, strict anaerobic incubation, and toxin detection assays should be incorporated in the future to ascertain the existence and clinical importance of this organism. The high levels of contamination observed in this study in Rosettenville resulted from improper storage practices that exposed dried fish to both flies and dust. The packaging methods at Sunnyside markets appeared to minimize pathogen exposure to food products. This aligns with the study by Siddhnath *et al.* [54], which shows that inadequate hygiene practices and improper storage methods lead to bacterial growth in dried fish products.

The presence of *S. aureus* in dried fish poses a major health risk because this bacterium causes contagious foodborne illnesses. Research findings indicate that *S. aureus* can survive on dried fish, as this product supports its salt tolerance and the temperature range of 30–37°C [57]. The presence of *Staphylococcus* in dried fish indicates inadequate handling processes among vendors [58]. Furthermore, the presence of *Staphylococcus* *lentus*, *S. sciuri,* and *S. saprophyticus* indicates the fish became contaminated by human activities. *Macrococcus. caseolyticus* was frequently detected in the collected samples due to its role as a spoilage agent. The microorganism appears primarily in environments where improper fish-handling practices are combined with exposure to market dust [59]. Studies confirm that *C. botulinum* resides in aquatic environments and in rotten seafood [60, 61], with researchers detecting this microorganism in smoked fish. The bacterial group *C. bifermentans* and *P. bifermentans* were detected in marine sediment samples, as they have potentially harmful effects on human health.

*Enterococcus faecalis,* together with *K. pneumoniae,* was detected and confirmed in this study, indicating fecal contamination that may originate from the vendors’ hands. The study by Ifedinezi *et al*. [62] demonstrated that, through potential water contamination or human contact, these bacterial species can contaminate foods and food products. The presence of *K. pneumoniae* in the collected samples in this study should be regarded as a public health concern, as *K. pneumoniae* poses major healthcare risks since it causes respiratory tract infections together with gastrointestinal illness. Studies confirm that processing methods and storage of dried fish products lead to the high detection frequency of *Enterococcus* spp. [63, 64]. This study found that smoked fish contained bacteria in 40.3% of specimens, as the smoking process allowed the fish to retain moisture. According to research [52, 65], microbial proliferation happens more easily in smoked fish when improper storage methods are used. Moreover, the results of this study reveal serious public health concerns due to the detection of *C. botulinum,* despite its low prevalence, as it produces botulinum neurotoxins, the most lethal biological toxin known [61, 65]. Spores of *C. botulinum* have been reported to germinate and release toxin in low-acid, high-protein foods, including dried, smoked, or salted fish, particularly when stored in anaerobic or poorly ventilated environments. Informal market environments, where temperature, packaging, and storage are generally substandard, can increase the likelihood of botulinum toxin formation. Although the study did not evaluate toxin production or gene expression, the occurrence of *C. botulinum* highlights the importance of improved processing and storage procedures, and of monitoring for high-risk pathogens in dried fish products, as it is a potential food safety hazard [49, 60].

### Phylogenetic analysis

The phylogenetic evaluation of dried fish bacterial isolates generated four distinct clusters using GenBank data on bacterial species. The bacterial isolates in Cluster 1 revealed a strong similarity (98%) between *K. pneumoniae* and *Klebsiella* spp., which grouped together with *S. xylosus*. Induction of *Macrococcus* *caseolyticus* revealed deviations from other bacterial strains present in the study. The Nigerian and South African *Staphylococcus* spp. and *L. macroides* isolates displayed a matching genetic relationship, as shown in Figure 5. *S. lentus,* along with *C. bifermentans* and *Enterococcus* spp., related to Japanese and GenBank strain records, belonged to Sub-group 2 of Cluster 2 (Figure 5). Cluster 3 showed high similarity to South African dairy isolates, as evidenced by the identification of *S. aureus* and *C. botulinum*. The genetic analysis in Cluster 4 links *S. aureus* to *Macrococcus* *caseolyticus,* which, as the data confirm, supports the decision to create a new independent genus [66]. Based on the phylogenetic relationships demonstrated in this study, dried fish contaminants pose potential health risks to humans. The phylogenetic clustering observed in this study shows bacterial relatedness between regional strains, such as South African dairy and fish isolates, offering strong insight into local transmission from an origin not commonly explored.

### Study limitations and future directions

In this study, a gap in bacterial detection and AMR panel was observed. Although this research has discovered several bacterial species including well known foodborne pathogens such as *S. aureus*, *K. pneumoniae*, *Clostridium*, and environmental flora we should admit that remarkably, generic food borne pathogens including *E. coli*, *Salmonella* spp., *Listeria monocytogenes*, and *Vibrio* spp., pertinent to fish and seafood microbiology, were not confirmed in this work because of the limitations of the culture based and the molecular targeting approach. These isolates could also have been present below the detection thresholds or not selectively enriched and would therefore have required specific molecular primers not incorporated into the current *16S rDNA *sequencing approach. Future investigations could involve species-specific PCR assays, next-generation sequencing, or metagenomic approaches that can extend pathogen coverage and uncover missing species or those that cannot be cultured. Likewise, the AMR panel used in this study comprised only seven antibiotics, focusing on commonly used agents such as streptomycin, erythromycin, amoxicillin, gentamicin, norfloxacin, chloramphenicol, and ciprofloxacin. Although informative, this panel lacks extended-spectrum β-lactams (e.g., cephalosporins), carbapenems, and tetracyclines, which are high priorities in AMR surveillance worldwide. Besides, the resistance gene profiling was not carried out, thereby reducing information on the genetic basis of resistance. Future studies are required that would include molecular detection of resistance determinants such as *bla*, *erm*, *tet,* or *mecA* genes, which would add to the general understanding of the resistance burden and its public health ramifications.

Various bacterial strains reacted differently to antibiotic examination with different antimicrobial agents. This study demonstrated that all *S. aureus* strains exhibited full resistance to erythromycin. This finding aligns with the resistance rates observed by Hu *et al*. [67] and Moges *et al*. [68]. Additionally, erythromycin resistance was observed in all *S. xylosus* strains, as well as in *S. sciuri*, *S. saprophyticus,* and *S. lentus*. These results completely align with Kim and Ahn [69], who show similar resistance patterns of antibiotic-resistant pathogenic food contaminants as recently documented. It was also observed in this study that 94% of *Staphylococcus* spp. subjected to antimicrobial tests were susceptible to chloramphenicol and norfloxacin. Furthermore, *S. aureus*, *S. xylosus*, *S. sciuri*, *S. saprophyticus* and *S. lentus* were susceptible to chloramphenicol; this aligns with studies [70, 71], demonstrating the effectiveness of chloramphenicol against Gram-positive bacteria. On the other hand, chloramphenicol, as well as amoxicillin and norfloxacin, produced complete susceptibility results in *Enterococcus* strains testing. In this study, all *E. faecium* and *E. faecalis* strains examined showed sensitivity to ciprofloxacin. The streptomycin and gentamicin resistance observed in the *E. faecalis* strains in this study reflects current patterns of resistance in *Enterococcus* spp. [72].

*Enterococcus* spp. usually cause no harm, but evidence demonstrates their association with intra-peritoneal and urinary tract infections [73]. This research demonstrated that selected *E. faecium* isolates were resistant to erythromycin, consistent with findings from studies of aquatic and foodborne bacteria. The research yielded contrasting results compared with previous reports on *E. faecalis* antibiotic response to amoxicillin and ampicillin, documenting high susceptibility rates and distinct antibiotic resistance patterns across regions [74, 75]. Study results showed that ciprofloxacin, gentamicin, and norfloxacin displayed effective susceptibility to *Klebsiella* spp. along with *K. pneumoniae,* whereas resistance to erythromycin and streptomycin was observed. The resistance of *K. pneumoniae* strains to streptomycin may result from an increasing number of ESBL- and carbapenem-producing strains [76]. Resistance of *K. pneumoniae* to ampicillin and erythromycin is increasing, yet chloramphenicol remains a viable treatment option [77–79]. The bacterium *Macrococcus* *caseolyticus* obtained from smoked fish displayed resistance to the antibiotics amoxicillin, streptomycin, and gentamicin. The presence of antibiotic-resistant bacteria in food products creates health dangers to public safety [80].

Based on the results of this study, we conclude that dried fish sold in informal markets around and within the sampling areas pose a serious public health concern that may contribute to foodborne disease among consumers. The microbiological quality of these dried fish sold in informal markets is unacceptable due to the presence of pathogenic bacteria of public health concern and their resistance to multiple antibiotics, thereby requiring improved measures to prevent contamination during processing. Relevant intervention methods, including surveillance, hygiene education, and alternative antimicrobial treatments, must be implemented in response to the increasing prevalence of multidrug-resistant bacteria. The research findings underscore the immediate need for food safety regulations in informal markets, as well as for antimicrobial stewardship. Furthermore, the identification of multidrug-resistant (MDR) bacteria in the isolates during this study is a major issue of food safety and population health. Table 11 presents an MDR profile by bacterial species, such as *Staphylococcus* spp., *K. pneumoniae*, *Clostridium* spp., and *Macrococcus* *caseolyticus*. The presence of MDR bacteria in dried fish products sold in the informal market implies that consumers may be exposed to pathogens that are challenging to treat with suitable antimicrobial agents. Further, the presence of MDR in food items such as dried fish may be regarded as a reservoir of resistance genes that can be transferred to humans via the food chain, adding to the overall AMR burden in the population and increasing the risk to the population’s health. This paper has shown that informal market environments with weak hygiene standards and regulatory measures are critical to the spread of MDR bacteria. Such results highlight the importance of improved food safety measures, enhanced food surveillance, and antimicrobial stewardship in the food production and distribution chain. Additionally, this study should be regarded as the first South African report to link dried fish from informal markets to multidrug-resistant strains, including *S. aureus*, *Klebsiella*, and *Enterococcus*, in a One Health context.

This study acknowledges certain limitations. Although the convenience sampling approach applied may be practical, it may reduce the generalizability of the findings from the sampled places. Also, the lack of random sampling leaves room for selection bias. The data obtained in this study reflect a particular moment, as seen in the non-seasonal differentiation of bacterial contamination and in vendors' practices. Besides, the absence of meticulous demographic profiling and lengthy vendor interviews limited the behavioral approach to factors influencing food safety. However, the findings provide an unequivocal understanding of microbial risks associated with dried fish in informal markets. Additionally, moisture content and water activity (a_w), which influence microbial survival in dried fish and toxin production by *C. botulinum*, were not measured in the current study and should be considered in future research that uses advanced molecular typing methods such as metagenomics. Additionally, data on consumer consumption patterns, including frequency and portion size of dried fish intake, were not collected, limiting the ability to estimate population-level exposure and public health risk.

## CONCLUSION

The present study assessed the food safety practices and microbial quality of dried fish sold in informal markets in the Gauteng Province, South Africa. Observational findings revealed widespread deficiencies in hygiene practices, including absence of PPE, poor environmental conditions (flies, stagnant water, dust, and proximity to waste), inadequate personal hygiene, and improper storage of products at ambient temperature. These conditions were consistent across most vending sites regardless of vendor age or gender.

Microbiological analysis showed high TBC across all three processing methods (salted, sun-dried, and smoked fish), with mean values ranging from 2.13 × 10⁷ to 2.91 × 10⁷ CFU/g. These levels substantially exceeded recommended benchmarks for dried or ready-to-eat fish products (10⁵–10⁶ CFU/g). Statistically significant differences were observed among fish types (p < 0.05), with sun-dried fish exhibiting the highest contamination, while no significant differences were found across sampling locations.

Molecular identification revealed a diverse bacterial profile dominated by *Staphylococcus* spp., *Clostridium* spp., *Klebsiella* spp., and *Enterococcus* spp., including species of public health concern such as *S. aureus*, *K. pneumoniae*, and *C. botulinum*. Many isolates exhibited MDR, with notable resistance to streptomycin and erythromycin. Phylogenetic analysis indicated genetic relatedness to regional and international strains, highlighting potential transmission pathways in informal market settings.

Overall, the findings indicate that dried fish sold in these informal markets pose a significant public health risk due to high microbial loads, presence of pathogenic and opportunistic bacteria, and widespread AMR. This study, considered the first such report linking dried fish from informal markets to MDR strains in a South African One Health context, underscores the urgent need for targeted interventions, including improved hygiene training, environmental controls, standardized processing and storage practices, enhanced surveillance, and antimicrobial stewardship in informal food value chains.

Future research should incorporate toxin detection, moisture/water activity measurements, broader pathogen panels using metagenomics, expanded AMR gene profiling, and consumer exposure assessments to better quantify and mitigate risks associated with these products.

## DATA AVAILABILITY

The supplementary data can be made available from the corresponding author upon request.

## GENERATIVE AI DECLARATION

The authors used QuillBot during the preparation of this manuscript solely for grammar, spelling, sentence structure, and language clarity. The tool was not used for the creation of scientific content, interpretation of data, analysis, conclusions or for the production of figures and tables. The authors, responsible for the content of the manuscript, had the final say in its development, review and approval, and in the accuracy and integrity of the scientific content.

## AUTHORS’ CONTRIBUTIONS

MM: Conceived and designed the study, secured funding, supervised the project, and reviewed the manuscript. SRN and MET: Collected the samples, conducted the laboratory investigations, and contributed to data acquisition. BGM, NL, and DL: Validated the laboratory procedures and results, interpreted the findings, and critically reviewed the manuscript. All authors have read and approved the final version of the manuscript.
